# scResponse: A Rank‐Based Method for Identifying Cell States That Contribute to Immunotherapy Response by Single‐Cell Data

**DOI:** 10.1002/advs.202524397

**Published:** 2026-03-23

**Authors:** Qi Dong, Haijun Huang, Yuxiu Wang, Bo Jiang, Yuzheng Chen, Huanhuan Xu, Jing Ren, Shengnan Guo, Tianhao Li, Mingjie Zhang, Tianyu Zhai, Wenbo Yang, Kaidong Liu, Cheng Lyu, Jingxuan Kang, Mingshuo Zhang, Yupeng Lu, Binghui Si, Shujun Cao, Dandan Xu, Zong Chen, Dapeng Hao

**Affiliations:** ^1^ Harbin Medical University Cancer Hospital Harbin Medical University Harbin China; ^2^ Key Laboratory of Molecular Oncology of Heilongjiang Province Harbin China; ^3^ School of Basic Medical Sciences Harbin Medical University Harbin China; ^4^ School of Intelligent Medicine and Technology Big Data Research Center Hainan Engineering Research Center for Health Big Data Hainan Medical University Hainan China; ^5^ Department of Hematology The Second Affiliated Hospital of Harbin Medical University Harbin China; ^6^ Department of Systems Biology College of Bioinformatics Science and Technology Harbin Medical University Harbin China; ^7^ Center for Endemic Disease Control Chinese Center for Disease Control and Prevention Harbin Medical University Harbin China; ^8^ National Health Commission Key Laboratory of Etiology and Epidemiology Harbin Medical University Harbin China; ^9^ Joint Key Laboratory of Endemic Diseases Harbin Medical University Guizhou Medical University Xi'an Jiaotong University Harbin China; ^10^ Department of Phase 1 Trials Center Harbin Medical University Cancer Hospital Harbin Medical University Harbin China

**Keywords:** cell state, immunotherapy, metabolism, single‐cell

## Abstract

Immunotherapy has shown great promise in cancer treatment, yet many patients fail to achieve long‐term efficacy. Single‐cell sequencing is a powerful technique for understanding how cellular diversity affects treatment outcomes; however, there remains a lack of robust methods for evaluating immunotherapy efficacy at single‐cell resolution. In this study, we present scResponse, an efficient algorithm that quantifies single‐cell responses to immunotherapy. scResponse captures subtle influences of diverse cellular subtypes and states on immunotherapy efficacy, including the effects of vascular normalization states of vascular cells, polarization of macrophages, terminal exhaustion of CD8^+^ T cells, and the opposing impacts from distinct fibroblast subtypes. By linking therapeutic response to single‑cell states, scResponse enables to delineate how distinct biological processes shape cellular states to drive divergent therapeutic outcomes. Integrated analysis across cancers identified key metabolic pathways associated with responsive states of tumor cells, including glyoxylate and dicarboxylate metabolism as immunotherapy‐sensitizing and retinol metabolism as suppressing it. As a proof‐of‐concept, the opposing roles of glyoxylate and retinol metabolism was validated using tumor‑CD8^+^ T cell co‐culture assays and in vivo mouse models. Thus, scResponse is an effective tool for mapping immunotherapy efficacy to single‐cell states, enabling mechanistic insights and target discovery for developing novel immunotherapy sensitization strategies.

## Graphical abstract

Although the tumor microenvironment is known to shape immunotherapy response, its key determinants remain elusive, underscoring the critical need for single‐cell resolution analytical frameworks. We present scResponse, an efficient algorithm to quantify single‐cell responses to immunotherapy. By capturing diverse cellular subtypes and states associated with treatment efficacy, scResponse provides a powerful tool for elucidating mechanisms underlying therapeutic response and resistance.

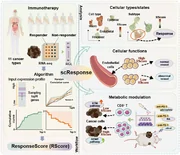

## 1 Introduction

Immune checkpoint blockade (ICB) has displayed great potential at the treatment of melanoma (MEL), non‐small cell lung cancer (NSCLC), metastatic renal cell carcinoma (RCC) and many other malignancies [1]. Despite their promise, patient response remains limited due to the complexity of tumor microenvironment (TME) [2]. This underscores the pressing need for computational tools capable of single‐cell resolution analysis for risk stratification and mechanistic studies. Current predictive tools for immunotherapy response primarily operate at the patient level, failing to capture the distinct contributions of diverse microenvironmental components [3]. For instance, identical PD‑1 expression levels carry different implications depending on whether they originate from CD8^+^ T cells, Regulatory T cells, germinal center B cells, or macrophages. The advent of single‑cell RNA sequencing (scRNA‐seq) overcomes the signal‑averaging limitation of bulk profiling and resolves cellular heterogeneity and dynamic states within TME, thereby enabling the identification of functional cell subsets critical to therapeutic efficacy [4, 5, 6].

Recently, several single‐cell based tools have emerged, such as scCURE that uses integrative CD8^+^ T cell features to predict immunotherapy outcomes in breast cancer, and PRECISE that leverages gene signatures across different cell types to improve phenotype prediction [7, 8]. In another study, Dong et al. employed pseudo‐bulk expression derived from scRNA‐seq for immunotherapy response prediction [9]. Although existing models leverage single‐cell data for feature extraction and training, they are designed to generate patient‐level predictions using pseudo‐bulk or bulk sequencing data. Consequently, they fail to capture the influence of individual cell states on immunotherapy or track per‑cell dynamic changes. To achieve single‑cell resolution, three key challenges must be addressed: (1) computationally efficiency scalable to hundreds of thousands of cells; (2) overcoming data sparsity and technical noise inherent to single‑cell profiles; and (3) enabling rapid model adaptation to user‑provided datasets.

In this study, we present scResponse, a rank‐based algorithm that rapidly infers cellular state associated with immunotherapy efficacy from user‐provided data. The rank‑based strategy has demonstrated high robustness to technical noise and dropout events inherent in single‐cell data, and remains effective across variable training cohort sizes [10, 11]. We utilized scResponse to quantify the differential contribution of cell types/states and identify cellular processes that affect immunotherapy response. Through pan‐cancer integrated analysis, we further identified critical metabolic pathways linked to tumor cell responsiveness, where glyoxylate and dicarboxylate metabolism (GDMT) enhances immunotherapy sensitivity while retinol metabolism (RTM) suppresses it. The opposing roles of these two pathways were experimentally validated via tumor‑CD8^+^ Tcell co‐culture and in vivo models, providing a proof‐of‐concept demonstration for the utility of scResponse analytical framework.

## 2 Results

### 2.1 Overview of the scResponse Framework

**FIGURE 1:**
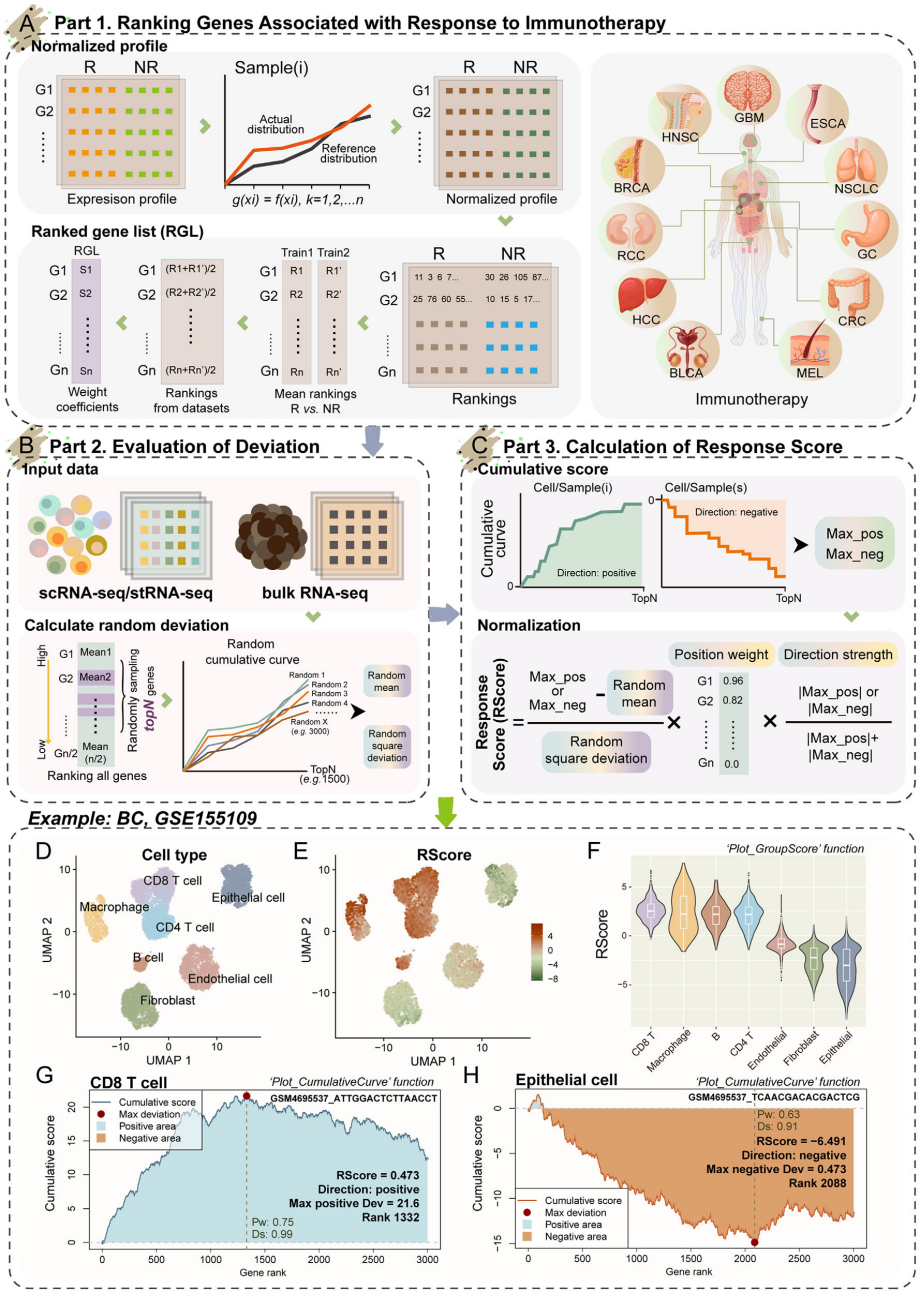
Overview of scResponse.(A) Part 1. Identification of the ranked gene list (RGL) associated with immunotherapy response. (B) Part 2. Calculation of Random_mean and Random_sd for the expected distribution via random sampling. (C) Part 3. Calculation of the Response Score (RScore) from an input expression matrix. (D–F) Distribution of RScores across cell types in the BC dataset (GSE155109). (G) Cumulative curve of a representative CD8^+^ T cell. (H) Cumulative curve of a representative epithelial cell.

The core concept of scResponse is to quantify the potential immunotherapy response of a single cell, assuming it captures a representative transcriptomic state of the tumor. This assumption enables rapid mapping between single‐cell states and patient‐level responses. To develop a scalable, rank‑based method capable of profiling large numbers of single cells, we collected bulk RNA‑seq data from 11 cancer types prior to immunotherapy and identified ranked gene lists (RGL) whose weights indicate relevance to immunotherapy response (Figure 1A). Expression data were normalized within each cancer type to remove scaling artifacts. Differential rank analysis between response (R) and non‑response (NR) patients was then performed to generate a cancer‑type‑specific RGL. Previously recognized biomarkers for immunotherapy response are prominently featured at the top of the RGL with weight coefficients > 0 (Figure S1A) [12, 13, 14, 15, 16]. With an RGL, an cumulative score would be easy to get for any given expression profile [17].

To account for potential positional bias within RGL, we first estimated a cumulative score distribution by simulating random selection of the top N genes based on expressed gene set in the scRNA‐seq data (Figure 1B). Then, a cumulative score for each cell was computed based on the ranking positions in RGL of its topN expressed genes. This score was then normalized against the background random variation to derive the final Response Score (RScore) for predicting response to immunotherapy (Figure 1C).

The patterns of RScore varied significantly across individual cells, especially those between different cell types (Figures 1D–F). In overall, immune cells such as CD8^+^ T cells displayed consistently higher scores, whereas epithelial cells generally exhibited lower scores. To facilitate interpretation, scResponse enables visualization of RScore distributions across predefined cell populations as well individual cells (Figure 1G,H).

We further explored the efficacy of RScore in predicting immunotherapy response. RScore demonstrated predictive performance with AUC values spanning from 0.6 to 1.0 across 25 immunotherapy bulk RNA‐seq datasets, and R samples exhibited significantly higher RScores than NR samples (Figure S2A,B). Patients with high RScore exhibited significantly longer overall survival (OS) and progression‐free survival (PFS) (Figure S2C–R). Additionally, spatial transcriptomic analysis revealed that spatial spots derived from immune‐hot exhibited significantly elevated RScore levels compared to those from immune‐cold (Figure S3A,B). Likewise, spots from R patients displayed significantly higher RScore than those from NR patients (Figure S3C–F). Collectively, our results demonstrate that RScore exhibits favorable predictive performance for immunotherapy response at the population level.

### 2.2 RScore Effectively Recapitulates the Differential Contributions of Various Cell Types and Cellular States to Immunotherapy Efficacy

**FIGURE 2:**
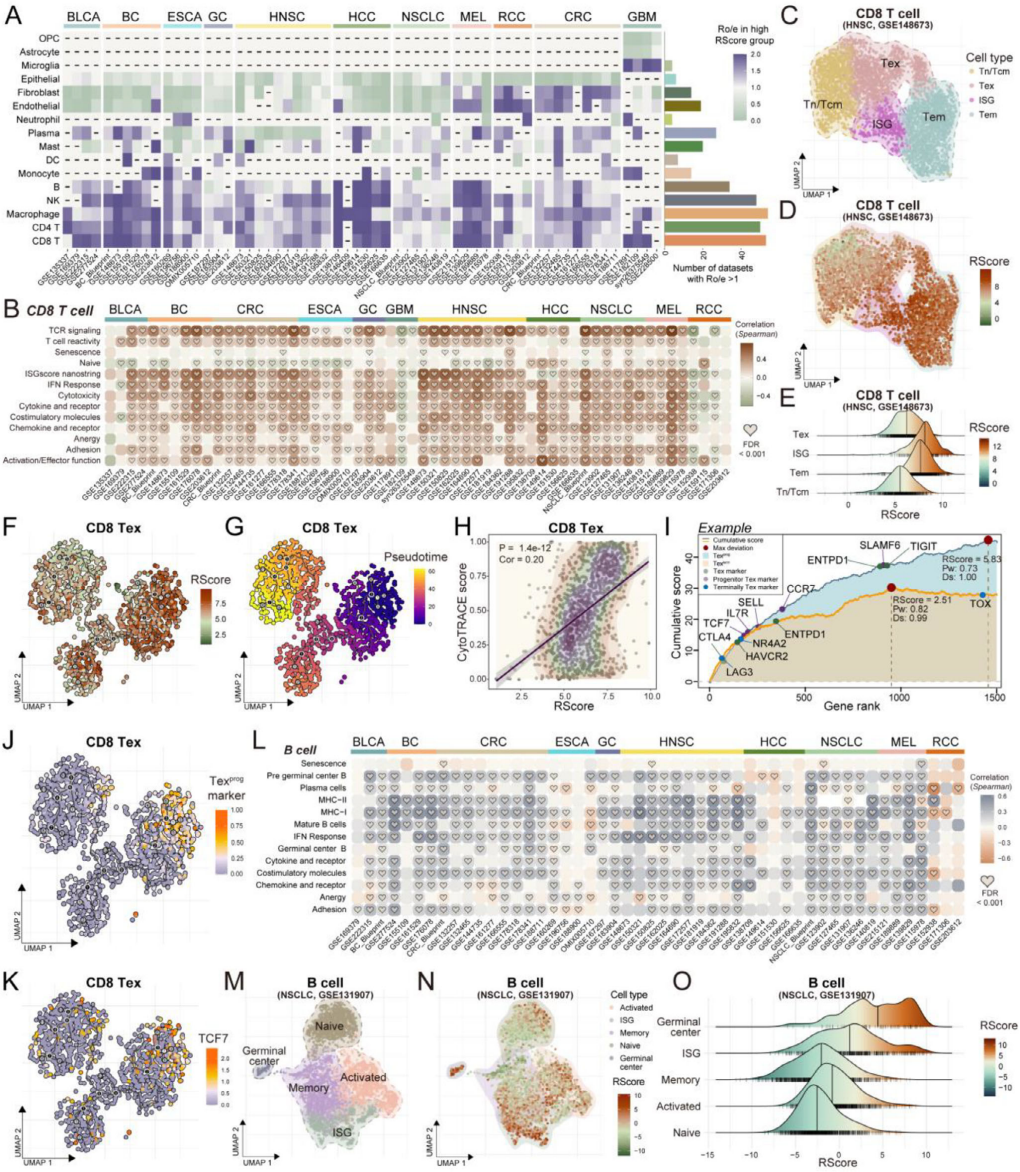
RScore reflects the differences in the contribution of CD8^+^ T and B cell subsets to immunotherapy.(A) The ratio of observed to expected (Ro/e) values of cell types that represent their enrichment in high RScore populations across pan‐cancer scRNA‐seq datasets. Cells were stratified into high RScore and low RScore groups based on the median value within the dataset. (B) Correlation between functional scores and RScores in CD8^+^ T cells. (C) Distribution of CD8^+^ T cell subtypes in the GSE148673 dataset. (D,E) Distribution of RScore across CD8^+^ T cell subtypes. (F) Distribution of RScores along the differentiation trajectory inferred by Monocle 3 for exhausted CD8^+^ T (Tex) cells. (G) Pseudotime developmental trajectory of CD8^+^ Tex cells. (H) Correlation between RScores and CytoTRACE differentiation scores during differentiation. (I,J) Expression of progenitor subpopulation markers and TCF7 along the differentiation trajectory. (K) Accumulated curves for a progenitor population of exhausted CD8^+^ T (Tex^prog^) and a terminally exhausted CD8^+^ T (Tex^term^). (L) Correlation between functional scores and RScores in B cells. (M) Distribution of B cell subtypes in the GSE131907 dataset. (N,O) Distribution of RScores across B cell subtypes. *P* values were calculated by Spearman's rank correlation analysis in (B), (H), and (L), *FDR* < 0.05 or *p* < 0.05 was considered statistically significant.

For the landscape overview of different cell types associated with immunotherapy efficacy, we analyzed 60 scRNA‐seq datasets spanning 11 cancer types (Figure S4A), and computed the RScore for each individual cell. The default topN of 1500 was selected based on the plateau of predictive performance (Figure S2A), stabilization of RScore correlation across single cells around 1500 (Figure S4B), and enrichment of known response signatures within this range (Figure S1). This threshold also approximates the average gene detection rate in scRNA‐seq datasets (Figure S4C), ensuring robustness and efficiency. We calculated the ratio of observed to expected (Ro/e) cell numbers to quantify the enrichment of cell types within high RScore populations (Figure 2A). Across most datasets, T cells and macrophages were enriched in high scoring groups, indicative of a positive association with immunotherapy efficacy. In contrast, epithelial cells were predominantly enriched in low scoring populations, suggesting that high epithelial content usually correlates with diminished therapeutic response. Notably, fibroblasts and endothelial cells exhibited variable RScore across cancer types, suggesting considerable heterogeneity in their functional states. We next analyzed cellular functions associated with RScore within each dataset. Genes upregulated in high RScore populations were significantly enriched in pathways that promote immune response, such as antigen processing and presentation (Figure S5, false discovery rate [*FDR*] < 0.05). Moreover, RScores showed a strong positive correlation with immune activation in T cells, such as cytotoxicity and activation/effector function, and a significant negative correlation with naive signals in most datasets (Figure 2B; Figure S6A, *FDR* < 0.001). These results indicate that RScore effectively captures the relationship between cell type specific immune activity and immunotherapy response.

To examine whether RScore could further delineate the functional distinctions of cell subpopulations in relation to immunotherapy response, we annotated CD8^+^ T cell subtypes and observed a dynamic RScore pattern across the differentiation continuum. Naïve/central memory (Tn/Tcm) cells displayed low scores, followed by elevated scores in effector memory (Tem) populations, which subsequently declined in exhausted (Tex) subsets (Figure 2C–E), indicating that RScore reflects the activation states of CD8^+^ T cells. We next examined the distribution of RScore across the Tex subpopulation. A developmental trajectory was reconstructed, revealing a progressive decline in RScore along the differentiation axis (Figure 2F,G). In line with this, RScore was significantly correlated with CytoTRACE differentiation scores (Figure 2H). Notably, the progenitor subset (Tex^prog^), previously implicated as a key responder to ICB, exhibited the highest RScore values among Tex cells (Figure 2I–K). This distinction, arising from the progressive silencing of immune related genes during terminal differentiation, was effectively captured at single‐cell resolution by RScore (Figure 2I). RScore enables the identification of key biological pathways influencing immunotherapy efficacy within a given cell type (Figure 2B,L). For example, in B cells, RScore strongly correlated with major histocompatibility complex (MHC) and interferon (IFN) response signals (Figure 2L, *FDR* < 0.001). In addition, the scores increased progressively from naïve to activated B cells, with germinal center B cells and interferon‑stimulated gene (ISG) B cells exhibiting the highest RScore values (Figure 2M–O; Figure S6B,C). The results indicate the ability of RScores to distinguish cell subpopulations with discrepant responsiveness to immunotherapy.

### 2.3 RScore Highlights Cell Types that Exhibit Substantial Heterogeneity in Their Potential Contribution to Immunotherapy

**FIGURE 3:**
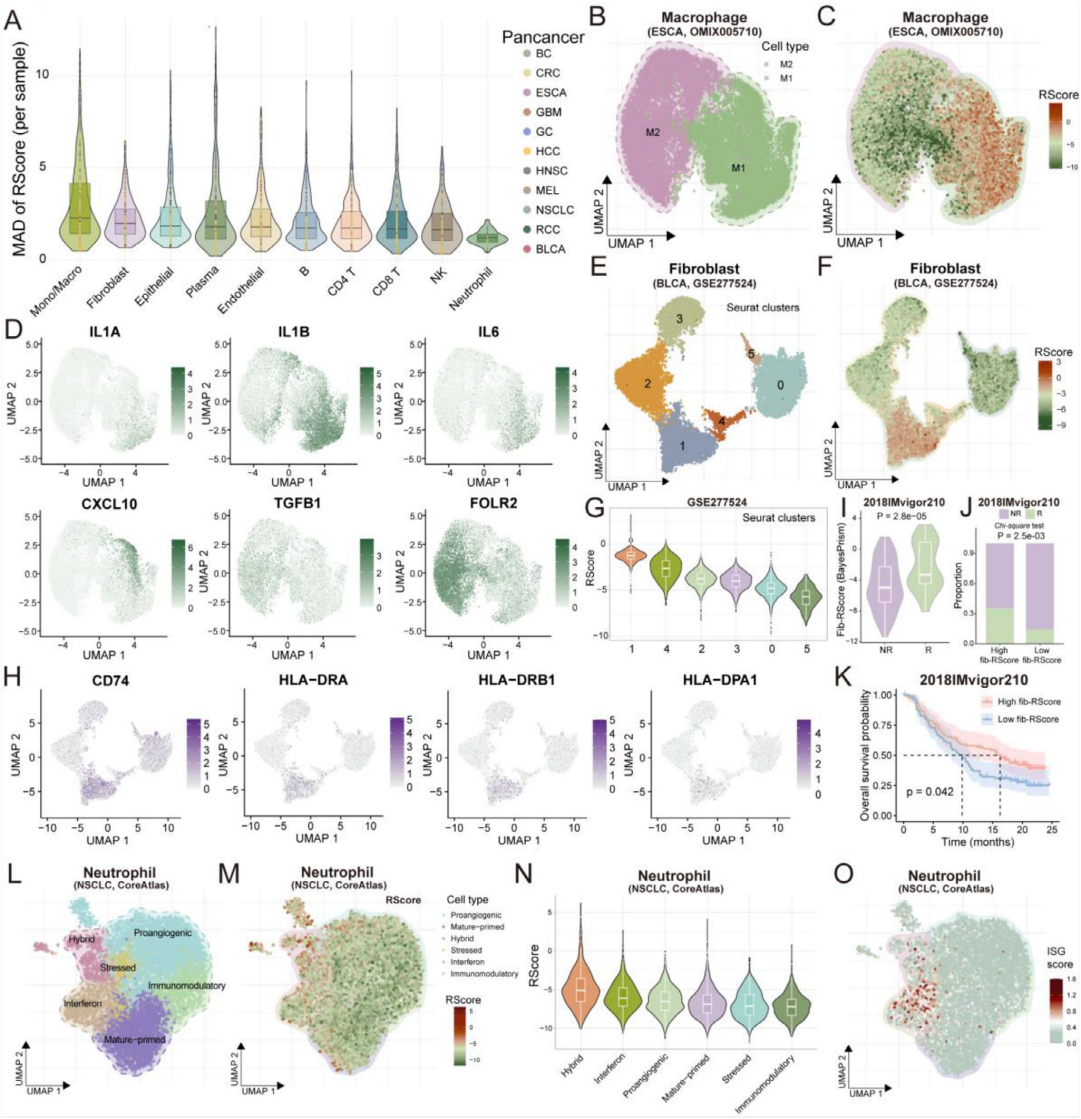
RScores identify cell subpopulations associated with response to immunotherapy. (A) The median absolute deviation (MAD) captures the within‐sample heterogeneity of each cell type in pan‐cancer scRNA‐seq datasets. (B,C) Distribution of RScores across macrophage subtypes in the OXIM005710 dataset. (D) The expression pattern of pro‐inflammatory M1 macrophage markers (IL1A, IL1B, IL6, and CXCL10) and anti‐inflammatory M2 macrophage markers (TGFB1 and FOLR2) of macrophages in the OXIM005710 dataset. (E–G) Distribution of RScore among fibroblast clusters identified via Seurat clustering in the GSE277524 dataset. (H) The expression pattern of antigen‐presenting caner associated fibroblast (apCAF) markers (CD74, HLA‐DRA, HLA‐DRB1, and HLA‐DPA1). (I) Fibroblast expression was inferred using a deconvolution approach, BayesPrism, and subsequently used to calculate fibroblast‐based RScore (fib‐RScore) via scResponse. The violin plot shows the fib‐RScore between response (R) and non‐response(NR) samples in the IMvigor210 dataset. (J) Differential distribution of R and NR samples between high fib‐RScore and low fib‐RScore groups in the IMvigor210 dataset. Patients were stratified based on the median fib‐RScore value. (K) Survival analysis for high and low fib‐RScore patients. (L–N) Distribution of RScores across neutrophil subtypes in the CoreAtlas dataset. (O) Distribution of interferon‐stimulated gene (ISG) scores across neutrophil subtypes in the CoreAtlas dataset. *P* values were calculated by one‐sided Wilcoxon rank‐sum test in (I), Chi‐square test in (J), and Log‐rank test in (L), *p* < 0.05 was considered statistically significant.

To assess the heterogeneity of RScore across tumor samples for each cell type, we calculated the median absolute deviation (MAD) of RScores for each cell type per sample across the 60 scRNA‑seq datasets (Figure 3A). Mono/Macro cells and cancer associated fibroblasts (CAFs) exhibited the highest RScore heterogeneity, indicating diverse functional states of different patients. Diversity in macrophages was primarily driven by polarization states, with FOLR2^+^ TGFB1^+^ macrophages typically exhibiting RScore < 0, whereas IL1^+^ macrophages showed RScore > 0 (Figure 3B–D; Figure S6D–F). CAFs were also segregated into RScore defined subpopulations reflecting opposing immunomodulatory functions (Figure 3E–G). The high RScore cluster, characterized by antigen‑presentation genes, in alignment with antigen‐presenting CAFs (apCAFs) that enhance anti‐tumor immunity (Figure 3H). In contrast, the low RScore cluster expressed PLAT/CXCL14, indicative of inflammatory CAFs (iCAFs) that drive immunosuppression (Figure S6G–J). This divergence underscores how CAF subtype composition differentially modulates immunotherapy outcomes, contributing to the high interpatient heterogeneity observed in therapeutic response. To further explore its association with immunotherapy response using RScore, we inferred the CAF transcriptomes in a bladder cancer immunotherapy dataset (IMvigor210) by deconvolution, and found that RScores of CAFs in responders were significantly higher than in non‐responders (Figure 3I,J; Figure S6K, *p* < 0.05). Furthermore, patients with higher RScores exhibited significantly improved prognosis (Figure 3K; Figure S6L), indicating that RScore not only identifies cell subtypes that promote immunotherapy response but also enables the evaluation of therapeutic efficacy at the level of individual cellular populations.

We next focused on neutrophils, which exhibit minimal interpatient variability yet differential states that mediate both pro‐ and anti‐tumor inflammation was also reported [18]. Our analysis revealed that anti‑tumor subtypes (e.g., hybrid and IFN‑responsive) neutrophils indeed exhibited significantly higher RScores than pro‑tumor subtypes (e.g., immunomodulatory and proangiogenic) (Figure 3L–O; Figure S7). This stable interpatient RScore pattern likely reflects conserved neutrophil states across patients. Collectively, these findings demonstrate that RScore effectively captures differential contributions of distinct cell types and states to immunotherapy response.

### 2.4 The RScore of Endothelial Cells Indicates Their Vessel Normalization State

**FIGURE 4:**
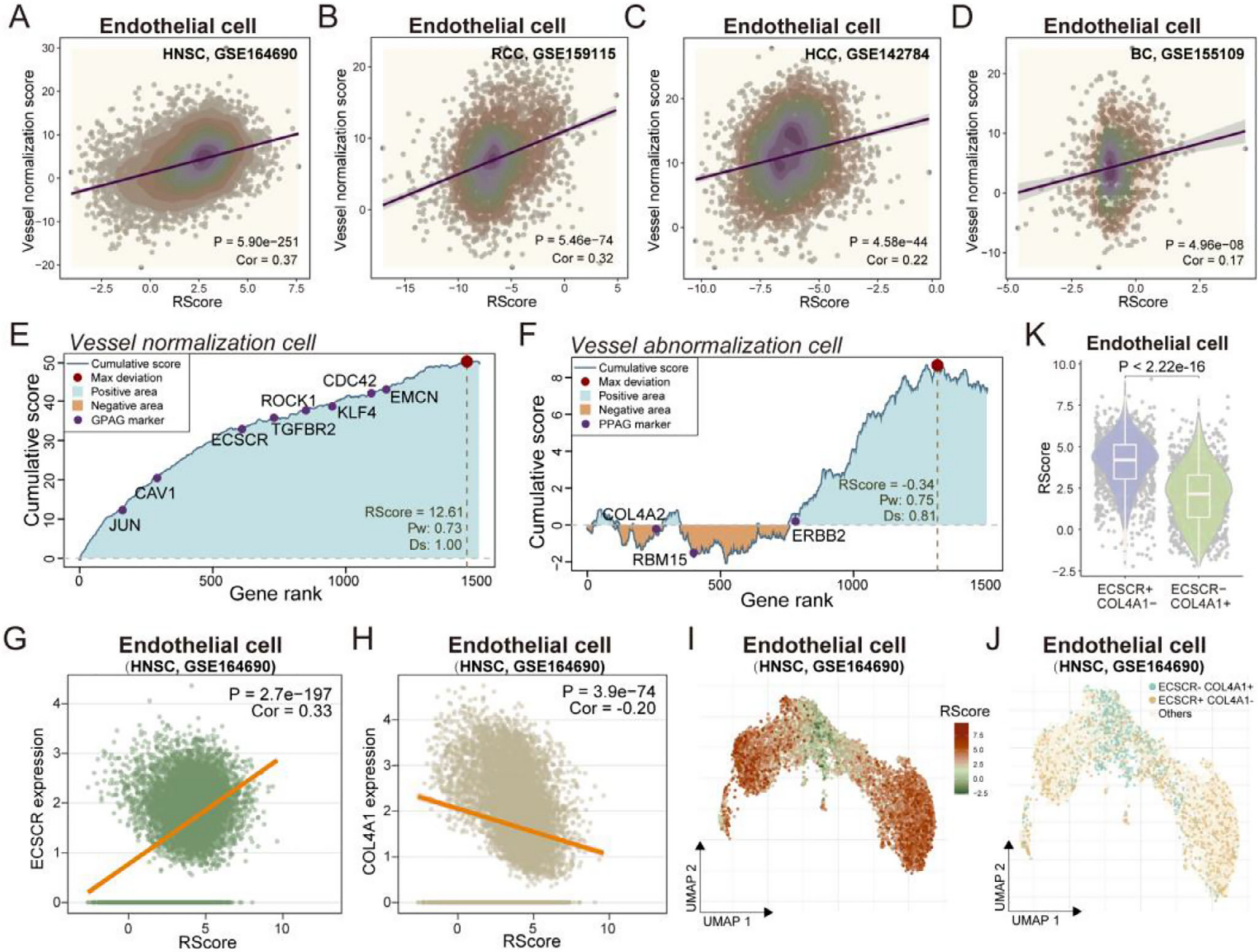
RScore positively associated with vessel normalization in endothelial cells. (A–D) Scatter plots showing the correlation between vessel normalization scores and RScores in endothelial cells. (E,F) Accumulated curves for a vessel normalized endothelial cell and a vessel non‐normalized endothelial cell. (G,H) Correlation between RScore and the expression of the good‐prognosis angiogenesis gene ECSCR and the poor‐prognosis angiogenesis geneCOL4A1. (I,J) Distribution of RScores in normalized (ECSCR^+^ COL4A1^−^) and abnormal (ECSCR^−^ COL4A1^+^) endothelial cells. (K) Differential distribution of RScores between ECSCR^+^ COL4A1^−^ and ECSCR^−^ COL4A1^+^ endothelial cells. *P* values were calculated by Spearman's rank correlation analysis in (A–D), (G), and (H), and by one‐sided Wilcoxon rank‐sum test in (K). *p* < 0.05 was considered statistically significant.

We observed considerable interpatient heterogeneity of RScore in endothelial cells (Figure 3A), with transcriptomic analysis pointing to vessel normalization that is known closely linked to immunotherapy efficacy by enhancing immune infiltration [19]. We found strong correlation between RScore and vessel normalization across multiple cancers (Figure 4A–D). High RScore endothelial cells expressed favorable angiogenic genes (e.g., ECSCR), while low RScore cells expressed poor prognosis markers (e.g., COL4A1; Figure 4E,F). These specific endothelial patterns highlight how RScore captures functional states linked to immunotherapy efficacy. Endothelial cells with high and low RScores showed high expression of normalized and abnormalized vascular genes, respectively (Figure 4E,F). Specifically, the expression of ECSCR and COL4A1 showed a significantly positive and negative correlation with RScore in endothelial cells, respectively (Figure 4G,H). In contrast, similar correlations were not observed in other cell types (Figure S8A,B). Furthermore, normalized endothelial cells (ECSCR^+^ COL4A1^−^) predominantly exhibited RScore > 0, whereas abnormalized cells (ECSCR^−^ COL4A1^+^) largely coincided with RScore < 0 (Figure 4I,J). Normalized subsets showed significantly higher RScores than abnormal subsets (Figure 4K). We next investigated the relative abundance of ECSCR^+^ COL4A1^−^ and ECSCR^−^ COL4A1^+^ endothelial cells in R versus NR group. The results showed that the fraction of ECSCR^+^ COL4A1^−^ cells was significantly higher in R than in NR samples, whereas ECSCR^−^ COL4A1^+^ cells were predominantly enriched in the NR samples (Figure S8C–E).

Notably, endothelial cells, though often a minor TME subset whose signals could be masked in bulk analyses, are effectively resolved by RScore at single‑cell resolution to reveal their subtle yet biologically critical contributions.

### 2.5 RScore Highlights the Difference of Epithelial Cells Between Responders and Non‐Responders of Immunotherapy

**FIGURE 5:**
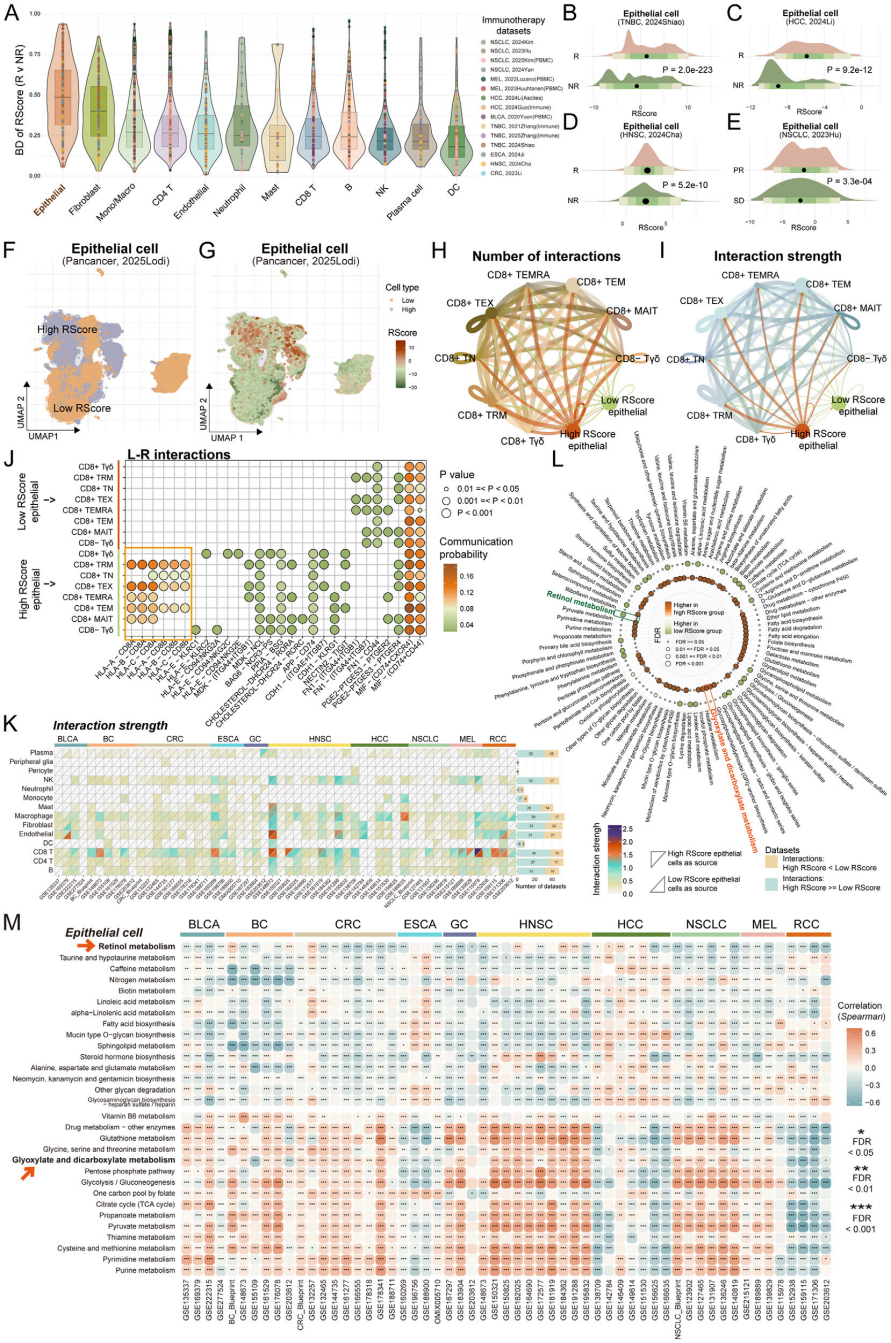
High RScore epithelial cells frequently communicate with CD8^+^ T cells. (A) Bhattacharyya distance (BD) values for each cell type computed between response (R) and non‐response (NR) samples across 15 immunotherapy scRNA‐seq datasets. (B–E) RScore distribution in epithelial cells from R vs NR samples in immunotherapy scRNA‐seq datasets. (F) Distribution of RScore groups in epithelial cells from seven cancer types in 2025Lodi dataset. Cells were grouped into high RScore and low RScore groups according to the sort for the median value of RScore in seurat clusters. (G) UMAP visualization of epithelial cells colored by RScore in the 2025Lodi dataset. (H,I) Circle plots showing cell‐cell communications among high and low RScore epithelial cells, and CD8^+^ T subtypes. Edge width represents the number and strength of interactions. (J) Significant ligand‐receptor interactions from high or low RScore epithelial cells to CD8^+^ T subtypes. (K) Strength of cell‐cell communications between high RScore (upper triangle) or low RScore (lower triangle) epithelial cells with other cell types across pan‐cancer scRNA‐seq datasets. Cells were stratified based on the median RScore value. Heatmap showing the inferred interaction strength. Blue bars indicate the number of datasets in which high RScore epithelial cells exhibited stronger communication than low‐RScore epithelial cells, yellow bars indicate the opposite. (L) Metabolic pathway enrichment in RScore‐stratified epithelial cells. Red and green dots indicate the enrichment levels in high and low RScore subpopulations, respectively. (M) Correlation between metabolic pathway enrichment scores and RScores in pan‐cancer scRNA‐seq datasets. The heatmap displays the top 15 positively and negatively correlated pathways across the majority of datasets. *P* values were calculated by one‐sided Wilcoxon rank‐sum test in (B–E), and (L), permutation test in (H–J), and Spearman's rank correlation analysis in (M). *FDR* or *p* < 0.05 was considered statistically significant.

When using the Bhattacharyya distance (BD) of RScore to identify cell types with the greatest divergence between immunotherapy responders and non‑responders, cancer (epithelial) cells exhibited the most pronounced difference (Figure 5A). In line with this, epithelial cells, fibroblasts and monocytes/macrophages in R samples showed significantly higher RScores than those in NR samples (Figure 5B–E; Figure S9). To explore functional differences, epithelial cells were stratified into high and low RScore groups using the pan‑cancer 2025Lodi dataset (Figure 5F,G; Figure S10A). Upregulated genes in the high RScore group were enriched for antigen processing and presentation pathway (Figure S10B, *FDR* < 0.05). High RScore epithelial cells displayed more frequent and stronger interactions with other cell types, particularly T cells and CD8^+^ T cell subtypes that are mediated by HLA ligand‑receptor pairs and MHC‑I signaling (Figure 5H–J; Figure S10C‑G). This enhanced communication pattern was conserved across independent pan‑cancer scRNA‑seq datasets (Figure 5K, S10H), suggesting that high RScore epithelial cells are more susceptible to ICB‐potentiated cell killing.

To identify pathways underlying the RScore labeled tumor cell states, we analyzed metabolic differences between high and low RScore groups. Across pan‑cancer datasets, metabolic pathways were segregated into two distinct clusters (Figure 5L,M, *FDR* < 0.05). Illustratively, we focused on GDMT, which was enriched the high RScore group and positively correlated with RScore in most datasets, and RTM, which was enriched in the low RScore group and negatively correlated with RScore. Consistently, the activity of GDMT was upregulated in responders, while RTM was downregulated in responders across different cancers (Figure S11A‐F). Moreover, GDMT positively correlated with immune infiltration, whereas RTM exhibited negative correlations with immune infiltration evaluated by multiple deconvolution methods (EPIC, CIBERSORT, TIMER) in TCGA cohorts (Figure S11G‑K). The results suggest that active GDMT likely promotes treatment response while elevated RTM appears to suppress it.

### 2.6 RScore Reveals Metabolic Modulation of Tumor Cell Response to Immunotherapy

**FIGURE 6:**
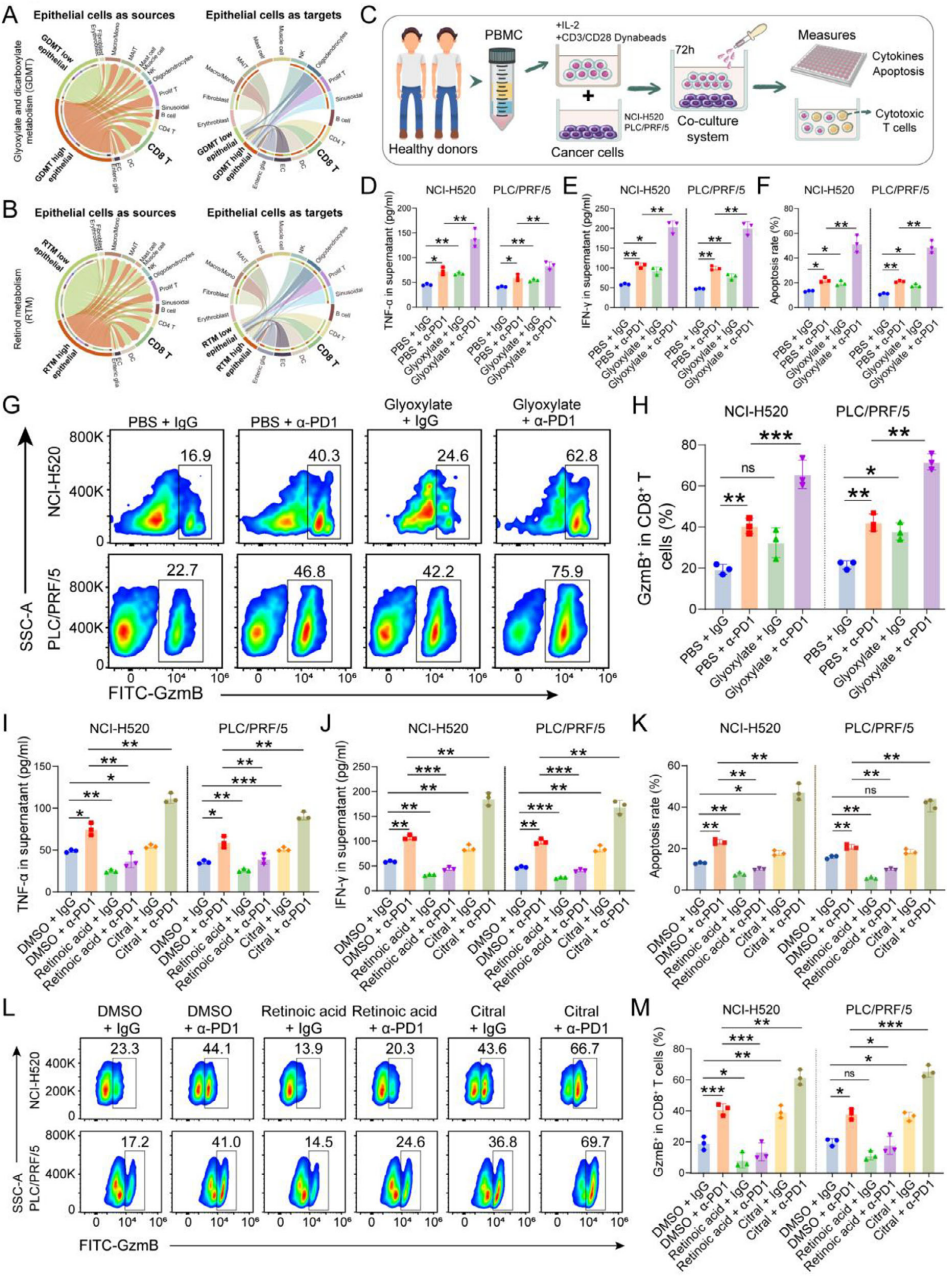
Altered GDMT and RTM of epithelial cells affects anti‐PD‐1 therapeutic efficacy in vitro co‐culture system. (A) The number of pathway signal that GDMT‐high and GDMT‐low sent (left) and received (right) with other cell types in pan‐cancer 2025Lodi dataset. Tumor cells were stratified based on the median of GDMT activities. (B) The number of pathway signal that RTM‐high and RTM‐low sent and received with other cell types in pan‐cancer 2025Lodi dataset. Tumor cells were stratified based on the median of RTM activities. (C) Schematic diagram of the co‐culture assay. (D,E) Concentrations of TNF‐α and IFN‐γ in culture supernatants determined by ELISA (*n* = 3). (F) Cytotoxicity assessed by LDH release assay (*n* = 3). (G,H) Representative flow cytometry graphs and quantification of GzmB^+^ cells in CD8^+^ T cells (*n* = 3). (I,J) Concentrations of TNF‐α and IFN‐γ in supernatants determined by ELISA (*n* = 3). (K) Cytotoxicity assessed by LDH release assay (*n* = 3). (L,M) Representative flow cytometry graphs and quantification of GzmB^+^ cells in CD8^+^ T cells (*n* = 3). Data are presented as Mean ± SD. Each dot in the bar graphs represents an individual sample. *P* values were calculated by permutation test in (A) and (B), two‐tailed Student's t‐test in (D–F), (H), (I–K), and (M). ^*^ *p* < 0.05; ^**^ *p* < 0.01; ^***^ *p* < 0.001. *p* < 0.05 was considered statistically significant.

We investigated the immune cellular subtypes whose interactions with tumor cells are most susceptible to perturbations in metabolic alterations of GDMT and RTM in tumor cells, thereby potentially modulating the anti‐tumor efficacy of immunotherapy. We grouped epithelial cells in the pan‑cancer 2025Lodi dataset by their median GDMT or RTM activities and performed CellChat analysis. Compared to GDMT‑low cells, GDMT‑high cells exhibited both increased incoming and outgoing communications with CD8^+^ T cells. Conversely, RTM‑high cells showed reduced interactions with CD8^+^ T cells relative to RTM‑low cells (Figure 6A,B). These results suggest that GDMT‑ and RTM‑associated metabolic states in epithelial cells may modulate immunotherapy efficacy by altering their communication with CD8^+^ T cells.

To evaluate the functional impact on immunotherapy efficacy, human peripheral blood CD8^+^ T cells were isolated and co‐cultured with cancer cell lines NCI‐H520 or PLC/PRF/5 (Figure 6C). Elevated levels of TNF‐α and IFN‐γ, and increased tumor cell apoptosis rates were observed in the co‐culture systems following combined glyoxylate and α‐PD1 antibody treatment (Figure 6D–F). Flow cytometry demonstrated that combined glyoxylate and α‐PD1 antibody treatment resulted in significantly higher proportions of GzmB^+^ CD8^+^ T cells compared to monotherapy (Figure 6G,H; Figure S12A). The effects of RTM to immunotherapy were explored by comparing retinoic acid (active metabolite of retinol), citral (an inhibitor of retinaldehyde dehydrogenase that blocks the conversion of retinol to retinoic acid), and combination of retinoic acid/citral and α‐PD1 antibody treatment in the co‐culture systems. Conversely, retinoic acid treatment alone or combined with α‐PD1 antibody decreased TNF‐α and IFN‐γ levels in co‐culture systems, and reduced apoptosis rate of cancer cells (Figure 6I–K). In contrast, citral treatment alone or combined with α‐PD1 antibody obviously increased TNF‐α and IFN‐γ levels, and apoptosis rate of cancer cells (Figure 6I–K). Furthermore, retinoic acid treatment alone or combined with α‐PD1 antibody significantly reduced the level of cytotoxic GzmB^+^ CD8^+^ T cells, whereas citral treatment alone or combined with α‐PD1 antibody significantly increased GzmB^+^ CD8^+^ T cells (Figure 6L,M). These experiments confirm that glyoxylate improves the efficacy of anti‐PD‐1 therapy in vitro, while retinoic acid exhibits inhibitory effect on anti‐PD‐1 therapy, which can be reversed by citral.

**FIGURE 7:**
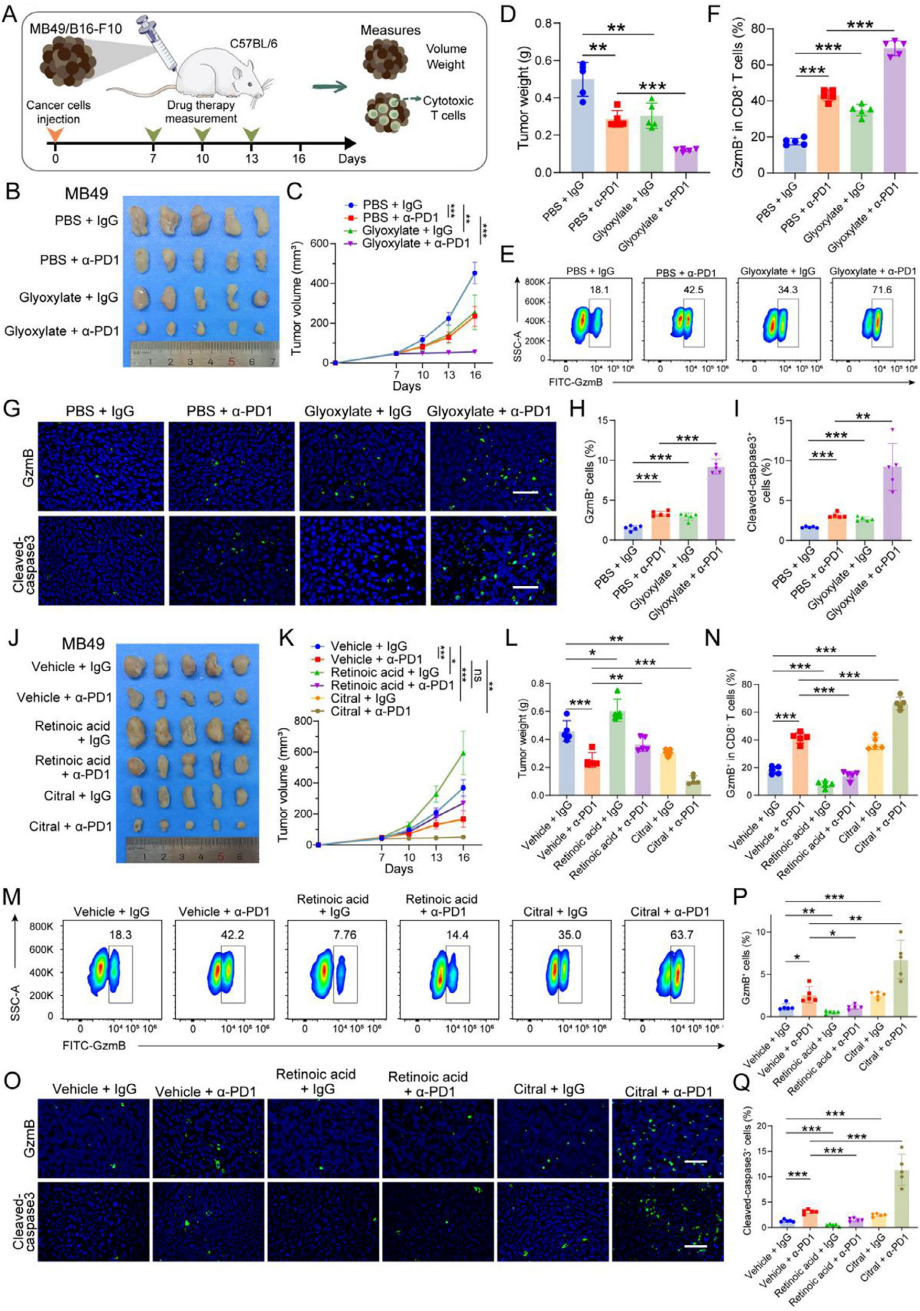
Altered GDMT and RTM of epithelial cells affects anti‐PD‐1 therapeutic efficacy in MB49 murine model. (A) Schematic illustration of the experimental design. (B) Representative gross images of excised tumors from MB49 tumor‐bearing mice (*n* = 5). (C) Tumor volume growth curves (*n* = 5). (D) Tumor weights at the endpoint (*n* = 5). (E,F) Representative flow cytometry graphs and quantification of the percentage of GzmB^+^ cells among CD8^+^ T cells within tumor‐infiltrating lymphocytes (*n* = 5). (G) Representative immunofluorescence images (scale bar, 50 µm) of GzmB and cleaved caspase‐3 staining in tumor sections (*n* = 5). (H,I) Quantification of the percentages of GzmB^+^ and cleaved caspase‐3^+^ cells (*n* = 5). (J) Representative gross images of excised tumors from MB49 tumor‐bearing mice with retinoic acid or citral combined with α‐PD1 treatment (*n* = 5). (K) Tumor volume growth curves (*n* = 5). (L) Tumor weights at the endpoint (*n* = 5). (M,N) Representative flow cytometry graphs and quantification of the percentage of GzmB^+^ cells among CD8^+^ T cells within tumor‐infiltrating lymphocytes (*n* = 5). (O) Representative immunofluorescence images (scale bar, 50 µm) of GzmB and cleaved caspase‐3 staining in tumor sections (*n* = 5). (P,Q) Quantification of the percentages of GzmB^+^ and cleaved caspase‐3^+^ cells (*n* = 5). Data are presented as Mean ± SD. Each dot in the bar graphs represents an individual subject. Statistical significance was determined by two‐tailed Student's *t*‐test unless otherwise stated. ^*^ *p* < 0.05; ^**^ *p* < 0.01; ^***^ *p* < 0.001.

We further investigated the effects of GDMT and RTM to immunotherapy efficacy in vivo using MB49 and B16‐F10 murine cancer cell lines (Figure 7A). In the MB49 and B16‐F10 subcutaneous tumor model, combination of glyoxylate and α‐PD1 antibody treatment significantly reduced tumor volumes and weights compared to glyoxylate or α‐PD1 antibody treatment alone (Figure 7B–D; Figure S12B–D). Flow cytometry analysis revealed that the combined treatment group show the highest proportion of tumor‐infiltrating GzmB^+^ CD8^+^ T cells in MB49 and B16‐F10 tumors (Figure 7E,F; Figure S12E–G). Moreover, immunofluorescence (IF) staining confirmed that the expression of GzmB and cleaved caspase‐3 was obviously increased by glyoxylate alone or combining with α‐PD1 antibody (Figure 7G–I; Figure S12H–J).

On the other hand, retinoic acid treatment increased the volumes and weights of MB49 subcutaneous tumors, which were decreased by citral, alone or combining with α‐PD1 antibody in both groups (Figure 7J–L). Flow cytometry showed that retinoic acid decreased the infiltration of GzmB^+^ CD8^+^ T cells, whereas citral increased the proportion of GzmB^+^ CD8^+^ T cells, a trend further amplified with the treatment of α‐PD1 (Figure 7M,N). Furthermore, IF staining confirmed the decreased level of GzmB and cleaved caspase‐3 by retinoic acid treatment, and their increased level by citral, both alone or combining with α‐PD1 (Figure 7O–Q). The above analyses further confirmed that glyoxylate enhances the efficacy of anti‐tumor immunity, especially under anti‐PD‐1 therapy, whereas retinoic acid suppresses therapeutic effect, and inhibition of retinoic acid by citral improves treatment outcomes.

## 3 Discussion

We developed scResponse, a scalable rank‐based algorithm designed to rapidly infer single‐cell responsiveness to immunotherapy across hundreds of thousands of cells. By efficiently calculating a straightforward RScore from cellular expression matrices, scResponse quantifies the contribution of distinct cell types and cell states to treatment outcome. scResponse provides RScore visualizations for both individual cells and cell populations, enabling users to assess the impact of cellular heterogeneity on immunotherapy response. Additionally, it enables the systematic identification of cellular functional variations associated with immunotherapy efficacy, offering a computationally efficient solution for profiling responsiveness across large single‑cell cohorts. In contrast to existing rank‐based methods (e.g., AUCell and ssGSEA), scResponse incorporates cancer‐specific RGL generation, response‐weighted gene prioritization, directional normalization, and dataset‐adaptive thresholding to mitigate zero inflation and enhance robustness across heterogeneous cohorts.

Utilizing scResponse, we identified key cell subpopulations contributing to immunotherapy response. For instance, ISG^+^ CD8^+^ T cells and Tex^prog^ CD8^+^ T cells, aligning with reports of their predictive and functional roles in treatment efficacy [20, 21, 22]. Similarly, pro‑inflammatory macrophages and apCAFs displayed high RScores, whereas immunosuppressive macrophages and iCAFs showed low RScores. ECSCR is a cell‐surface protein selectively expressed by endothelial cells. It coordinates cell adhesion, migration, and growth factor receptor signaling, and contributes to signal transduction pathways critical for vascular homeostasis [23, 24]. Mutations in COL4A1 are associated with genetically dominant vascular abnormalities and cause defects in vascular function [25, 26]. We show that the normalized (ECSCR^+^ COL4A1^−^) endothelial cells exhibited higher RScore than abnormalized (ECSCR^−^ COL4A1^+^) endothelial cells. These results illustrate how RScore captures functional dichotomies across diverse cellular subsets within the TME. The masking of minor but critical cell populations in bulk analyses underscores that averaging across the TME obscures functionally decisive signals. Our work posits that single‐cell resolution not just increases in granularity but a fundamental requirement for accurately mapping the cellular ecosystem that determines treatment success or failure. Previously unknown cell subpopulations associated with immunotherapy response may be uncovered by applying scResponse in further exploration.

Metabolic interventions hold promise for improving the effectiveness of immunotherapies [27]. scResponse enables the systematic identification of various metabolic pathways associated with treatment response. As representative examples, GDMT and RTM were identified to promote and attenuate the therapeutic effect, respectively. These predictions were supported by co‑culture and in vivo experiments. Previous studies demonstrated that RA suppresses anti‐tumor immunity by modulating certain immune cells, such as by promoting immunosuppressive macrophages and reducing dendritic cells and natural killer cells [28, 29, 30]. In alignment with the previous reports, we showed that RA suppresses anti‐PD‐1 efficacy by reducing cytotoxic CD8^+^ T cell activity. Our RScore‑based analysis further suggests that retinoic acid may also directly induce an immune‑resistant state in tumor cells themselves. These results demonstrate that scResponse can uncover key functional pathways underlying immunotherapy response and resistance. Future functional studies are poised to validate and expand this catalog of targetable metabolic modulations of immunotherapy.

scResponse does not require paired samples and is not limited to immunotherapy contexts, thereby enabling the identification of cell subpopulations associated with treatment response in any single‑cell dataset. In the future, we will continue to refine this cancer‐specific scoring system, including optimizing the feature selection algorithm to improve sensitivity for rare but critical cell states, and developing an ecosystem level risk‐stratifying tool by leveraging RScore‐based TME profiling. In summary, as a tool that dynamically maps patient‐level response features to single‐cell resolution, scResponse captures critical TME characteristics associated with immunotherapy, and provides insights into the potential resistance mechanisms. scResponse will facilitate the development of more precise, effective and broadly suitable tumor immunotherapeutic strategies.

## 4 Experimental Section

### 4.1 scResponse Pipeline Construction

Bulk transcriptomics data for 11 cancer types (NSCLC, MEL, HCC, BLCA, RCC, BC, GC, ESCA, HNSC, GBM, and CRC) prior to immunotherapy were obtained (Table S1).

Step 1. Generation of cancer‐specific RGL. Quintiles for each sample in training expression profile were calculated for generating reference distribution, the normalized profile were computed by linear fractal interpolation function. Then, genes were ranked in each sample according to the normalized values. For each gene, the difference in mean ranking values between R (CR+PR) and NR (PD) samples was termed the response‐related ranked weight coefficient, which was subsequently averaged over multiple datasets to yield the final coefficient. The RGL in a cancer type was composed by all ranked weight coefficients for genes in training datasets.

Step 2. Calculation of the random deviation for input expression profile. For each input expression profile (e.g., scRNA‐seq, stRNA‐seq, or bulk RNA‐seq), genes were ranked in descending order based on their mean expression values across all samples. TopN genes were randomly extracted from top 1/2 or 1/3 expressed genes through 3,000 iterations. In each iteration, the cumulative sum was calculated, and the maximum deviation (Max_dev), defined as the difference between maximum positive cumulative score (Max_pos) and minimum cumulative score (Max_neg), was confirmed every time. This random procedure yielded the mean (Random_mean) and standard deviation (Random_sd) of all Max_dev for the given expression profile.

Step 3. Calculation of the RScores for input expression profile. For each cell or sample i, the cumulative sum for genes belonging to the RGL that appeared within the top N genes were calculated. The cumulative score was then adjusted and normalized using the position weight (Pw) and direction strength (Ds) according to the following procedure:

Pw(i)=1−Max_pos(i)nGenes2Ds(i)=Max_pos(i)Max_pos(i)+Max_neg(i)Ds(i)=(ifMax_pos(i)>=Max_neg(i))or,Ds(i)=Max_neg(i)Max_pos(i)+Max_neg(i)Ds(i)=(ifMax_pos(i)<Max_neg(i))Normalized_score(RScore)(i)=Max_dev(i)−Random_meanRandom_sd×Pw(i)×Ds(i)

We have encapsulated the aforementioned computational workflow in R package “scResponse” for public use. Users could obtain the RScores by calling the function ‘Response_Score’ with the input expression profile and specified top N genes (default value is 1500). The ‘Plot_GroupScore’ function enabled users to visualize the inter‐group distribution of RScores. The ‘Plot_CumulativeCurve’ function visualized the cumulative curve for individual cell or sample. In this study, we calculated RScore with topN = 1500. If not specified, cells or samples were grouped by the median of RScore.

### 4.2 Isolation and Co‐Culture of Human CD8^+^ T Cells

Peripheral blood mononuclear cells (PBMCs) were isolated from the peripheral blood of three healthy donors using Human Lymphocyte Separation Medium (Ficoll) (Solarbio, Cat# P8610). CD8^+^ T cells were subsequently purified using a negative selection magnetic bead kit (BeaverBeads, Cat# 22204). Freshly isolated T cells were activated with CD3/CD28 Dynabeads (Thermo Fisher Scientific, Cat# 11131D) and cultured in RPMI‐1640 medium supplemented with 10% heat‐inactivated FBS (Gibco, Cat# 10099–141), 10 ng/mL IL‐2 (BioLegend, Cat# 589102), 10 mm HEPES (Beyotime, Cat# ST090), 2 mM Glutamine (Beyotime, Cat# C0212), 0.1 mM MEM Non‐Essential Amino Acids (Beyotime, Cat# C0208), 1 mm Sodium Pyruvate (Beyotime, Cat# C0218), and 50 µm 2‐Mercaptoethanol (Beyotime, Cat# M1210).

For co‐culture assays, NCI‐H520 or PLC/PRF/5 tumor cells were seeded at a density of 5 × 10^4^ cells per well in 48‐well plates. After 24 h of adherence, 2 × 10^5^ activated human CD8^+^ T cells were added to each well. The co‐culture system was treated with 10 µg/mL anti‐human PD‐1 antibody (Clone EH12.2H7; BioLegend, Cat# 329926) or isotype control (Clone MOPC‐21; BioLegend, Cat# 400166), in the presence of 100 µm Sodium glyoxylate (Aladdin, Cat# S339510), 100 µm Retinoic acid (Solarbio, Cat# A9120), or 100 µm citral (Aladdin, Cat# C104134). After 72 h of co‐culture, cells were collected to analyze the proportion of GzmB^+^ CD8^+^ T cells by flow cytometry. Concurrently, supernatants were harvested to assess cytotoxicity using an LDH Release Assay Kit (Beyotime, Cat# C0017) and to quantify TNF‐α (Proteintech, Cat# KE00068) and IFN‐γ (Proteintech, Cat# KE00063) levels using ELISA kits, strictly following the manufacturer's instructions.

### 4.3 Cell Culture

The human lung squamous cell carcinoma cell line NCI‐H520 (RRID:CVCL_1566) and the murine bladder carcinoma cell line MB49 (RRID:CVCL_7076) were purchased from Wuhan Sunncell Biotechnology Co., Ltd. (Wuhan, China). The murine melanoma cell line B16‐F10 (RRID:CVCL_0159) and the human hepatoma cell line PLC/PRF/5 (RRID:CVCL_0485) were obtained from the Cell Bank of the Chinese Academy of Sciences (Shanghai, China). All cell lines were authenticated by Short Tandem Repeat (STR) profiling and were confirmed to be free of mycoplasma contamination using PCR‐based detection assays. B16‐F10 and MB49 cell lines were cultured in DMEM (Gibco, Cat# 11995065) supplemented with 10% heat‐inactivated FBS (Gibco, Cat# 10099‐141) and 1% penicillin‐streptomycin (Beyotime, Cat# C0222). NCI‐H520 was maintained in RPMI‐1640 medium (Gibco, Cat# 11875093) containing 10% FBS and 1% penicillin‐streptomycin. PLC/PRF/5 was cultured in MEM (Gibco, Cat# 11095080) supplemented with 10% FBS and 1% penicillin‐streptomycin. All cells were maintained in a humidified incubator at 37°C with 5% CO^2^.

### 4.4 Tumor Models and Treatment

All animal experiments were approved by the Ethics Committee of Harbin Medical University (No. HMUIRB2024025). C57BL/6 female mice were inoculated subcutaneously on the flank with 1 × 10^6^ MB49 cells or 5 × 10^5^ B16‐F10 cells. Following tumor inoculation, mice were randomized into treatment groups. On days 7, 10, and 13 post‐inoculation, mice received intraperitoneal (i.p.) injections of 200 µg anti‐mouse PD‐1 antibody (Clone RMP1‐14; Selleck, Cat# A3990) or Rat IgG2a isotype control (Clone 2A3; Selleck, Cat# A3902). Concurrently, mice were intraperitoneally injected with Sodium glyoxylate monohydrate (40 mg/kg; Aladdin, Cat# S339510; dissolved in PBS), Retinoic acid (40 mg/kg; Solarbio, Cat# A9120; dissolved in 5% DMSO + 95% corn oil), citral (40 mg/kg; Aladdin, Cat# C104134; dissolved in 5% DMSO + 95% corn oil), or the corresponding vehicle controls. Tumor dimensions were measured every three days using calipers, and tumor volume was calculated using the formula: V = (L × W^2^)/2, where L is the length and W is the width. On day 16, mice were euthanized in accordance with institutional ethical guidelines, and subcutaneous tumors were harvested for subsequent experiments.

### 4.5 Pan‐Cancer Single‐Cell Transcriptomic Data Processin

A total of 60 scRNA‐seq datasets for 11 cancer types were obtained from public resources (Table S2). The scRNA‐seq data from each study was processed using R package Seurat, and integrated cells across individuals using integrate cells across individuals. Cell clusters were identified via the FindClusters function, and the clusters were annotated by canonical cell markers.

Lodi et al. presented a single‐cell atlas by aggregated 230 treatment‐naive samples across nine cancer types (Pancancer, 2025Lodi) [31]. We identified clusters for 100076 cancer/epithelial cells by Seurat, and grouped them into high and low RScore subpopulations according to the median. Salcher et al. aggregated 29 publicly available datasets to construct a NSCLC single‐cell atlas (NSCLC, CoreAtlas) [18]. Neutrophils were extracted and annotated into subpopulations according to the canonical markers [32].

We collected 15 scRNA‐seq data of ICB immunotherapy treatment for cancer samples (Table S3). RScore were computed for cells prior to immunotherapy. Cells derived from CR or PR patients were considered as response group, which derived from stable disease or PD patients were considered as non‐response group. The RScores for three publicly available stRNA‐seq datasets were calculated (Table S4).

### 4.6 Bulk RNA‐seq and Deconvolution

Gene expression profiles of The Cancer Genome Atlas (TCGA) were obtained from https://portal.gdc.cancer.gov/. Utilizing the BLCA scRNA‐seq (GSE27544) as a reference, we employed R package BayesPrism and CIBERSORTx (https://cibersortx.stanford.edu/) to infer the fibroblast composition and transcriptomes within the 2018IMvigor210 immunotherapy dataset [33]. The fib‐RScores were inferred for patients using scResponse.

### 4.7 Pseudotime Trajectory Analysis

The CytoTRACE algorithm was used to estimate the differentiation (stemness) status of epithelial cells or tumor cells using R package CytoTRACE [34]. Cells were given a CytoTRACE score indicating differentiation potential, with higher score signifying poor differentiation.

R package monocle3 was used for dividing different cell clusters into different differentiation partitions on the basis of potential differentiation relationships [35]. We used a simple principal tree algorithm simplePPT to construct individual trajectories and calculate pseudotime values, achieving simultaneous construction of multiple differentiation trajectories. The root nodes were specified according to CytoTRACE scores.

### 4.8 Cell‐Cell Communication Analysis

Cell‐cell communications were inferred using R package CellChat [36]. We focused on the human database in CellChat, identified significant cell‐cell communication by assigning probability values to each interaction and performing permutation tests. Due to huge number of cells in 2025Lodi dataset, we randomly sampling 2000 high and low RScore subpopulations respectively to infer cell‐cell communications for 10 times, the final communications were average.

### 4.9 Metabolism Analysis

scMetabolism was a computational pipeline for quantifying single‐cell metabolism [37]. We utilized R package scMetabolism to evaluate the metabolic pathway activity of epithelial cells or tumor cells based on AUCell quantification method. The 85 KEGG metabolism pathways were obtained from scMetabolism and used to compute the metabolism score using Gene Set Variation Analysis (GSVA) method with R package GSVA for bulk transcriptome data.

### 4.10 Functional Analysis

The functional gene sets about CD8^+^ T, CD4^+^ T and B cells were acquired from Lodi et al. The genes for ISGscore nanostring were obtained from Pelka et al. Signature of pre germinal center B and germinal center B are from Yang et al. and Ma et al. We evaluated the functional scores for cells using R package AUCell [38]. Tian et al. defined (∑GPAGs‐∑PPAGs) as a vessel normalization indicator. we evaluated vessel normalization level for endothelial cells. All the above gene sets are listed in Table S5.

### 4.11 Evaluation of RScore Variation

The MAD values for cell types across samples were calculated using mad function in R software. To evaluate the variation of RScore between R and NR samples, the BD were calculated by dividing RScore into bins for discretization in immunotherapy scRNA‐seq datasets.

### 4.12 Statistics

The one‐sided Wilcoxon rank‐sum test was applied for comparisons between two groups. Spearman's rank correlation analysis was utilized to compute the association between two variables. The chi‐square test was used to assess the categorical difference between groups. All data were presented as mean ± standard deviation. Student's t test was used to compare the mean values of independent samples. Statistical analyses were conducted using R software (https://www.r‐project.org) and GraphPad Prism software (https://www.graphpad.com/scientific‐software/prism/). *p* value < 0.05 was considered statistically significant. *P* values were adjusted using the Benjamini–Hochberg procedure to control the *FDR*. See Supplementary Files for further details and abbreviations.

## Author Contributions

Q.D. and H.H. contributed equally to this work. The research study was designed by D.H. Data were collected by Q.D., M.Z., T.Z., W.W., and K.L., while Q.D., H.H., Y.W., J.R., and H.X. performed the data analysis. Q.D., H.H., J.R., Y.C., S.G., T.L., J.K., M.Z., Y.L., and B.S. ran software. The analysis was validated by Q.D., H.H., Y.W., J.B., S.G., and T.L. The analysis was visualized by Q.D., H.H., Y.C., H.X., and S.C. Additionally, D.H., Z.C., and D.X. conceptualized the idea for the study. Funding was acquired by D.H. and Q.D. Q.D. and H.H. wrote original draft. The study was supervised, put forward methodology, supervised, reviewed, and edited by D.H. The authors read and approved the final manuscript.

## Conflicts of Interest

The authors declare no conflicts of interest.

## Supporting information




**Supporting File 1**: advs74902‐sup‐0001‐SuppMat.docx.

**Supporting File 2**: advs74902‐sup‐0002‐TablesS1‐S5.xlsx.
[Correction added on 20 April 2026 after first online publication: supporting information file has been updated in this version.]

## Data Availability

“scResponse” is an available tool on GitHub (https://github.com/dongqi777/scResponse). No new data were generated or analyzed in support of this research. All data analyzed during this study can be downloaded from public data resources, are described in the “Experimental Section”. Other information was provided in the Supplementary files.
