# Supplementary material for: scResponse: A Rank‐Based Method for Identifying Cell States That Contribute to Immunotherapy Response by Single‐Cell Data
Source: Adv Sci (Weinh). 2026 Mar 23;13(31):e24397. doi: 10.1002/advs.202524397 (PMC13588071; doi:10.1002/advs.202524397)
Supplement: Supplementary file 1 [file ADVS-13-e24397-s002.docx]

**scResponse: a rank-based method for identifying cell states that contribute to immunotherapy response by single-cell data**

**Contents**

[1. Supplemental Methods	3](#_Toc446)

[1.1 Pan-cancer scRNA-seq data processing	3](#_Toc29210)

[1.2 Spatial transcriptome RNA sequencing data processing	4](#_Toc1505)

[1.3 Ro/e analysis	5](#_Toc31145)

[1.4 Cell-cell communication analysis	5](#_Toc14313)

[1.5 Functional analysis	5](#_Toc31632)

[1.6 Receiver operating characteristic curve analysis	6](#_Toc5084)

[1.7 Survival analysis	6](#_Toc17892)

[1.8 Immunofluorescence staining	7](#_Toc4345)

[1.9 Flow cytometry analysis of tumor-infiltrating lymphocytes	7](#_Toc22115)

[1.10 Flow cytometry of cocultured T cells	8](#_Toc29591)

[2. Supplemental Figures	9](#_Toc28499)

[Figure S1. The ranked gene list for pan-cancer](#_Toc28428)*[.](#_Toc28428)*	[9](#_Toc28428)

[Figure S2. Association of the RScore with immunotherapy response and patient survival	10](#_Toc4171)

[Figure S3. Spatial distribution of RScore in stRNA-seq datasets	11](#_Toc8126)

[Figure S4. Distribution of nFeature_RNA and variation of RScores in pan-cancer scRNA-seq datasets](#_Toc23171)*[.](#_Toc23171)*	[12](#_Toc23171)

[Figure S5. KEGG functional enrichment analysis](#_Toc26316)*[.](#_Toc26316)*	[13](#_Toc26316)

[Figure S6. Annotation of cell subtypes and bulk RNA-seq deconvolution	14](#_Toc25686)

[Figure S7. Annotation of neutrophil subtypes	15](#_Toc15348)

[Figure S8. Correlation of expression of ECSCR and COL4A1 with RScore and immunotherapy response	16](#_Toc24090)

[Figure S9. Fibroblasts and monocytes/macrophages derived from responders exhibit high RScores](#_Toc28567)*[.](#_Toc28567)*	[17](#_Toc28567)

[Figure S10. Differential cell-cell communications of high and low RScore epithelial cells with other cell types.	18](#_Toc14976)

[Figure S11. Correlation of metabolic pathway and immunotherapy outcome and immunotherapy infiltration	19](#_Toc26136)

[Figure S12. Glyoxylate combined with anti-PD-1 therapy significantly suppresses B16-F10 melanoma growth.	20](#_Toc24334)

[3. Supplemental Tables	22](#_Toc24286)

[Table S1. Bulk transcriptomics data of cancer samples that prior to immunotherapy.	22](#_Toc17795)

[Table S2. Pan-cancer scRNA-seq datasets.	22](#_Toc11462)

[Table S3. ScRNA-seq data of cancer samples that prior to immunotherapy.	22](#_Toc32022)

[Table S4. Single-cell spatial transcriptomic datasets.	22](#_Toc13003)

[Table S5. Functional gene sets and cell markers.	22](#_Toc13040)

[4. Abbreviations	23](#_Toc20257)

[5. References	25](#_Toc25438)

1. Supplemental Methods

**1.1 Pan-cancer scRNA-seq data processing**

A total of 60 scRNA-seq datasets for 11 cancer types were obtained from public resources (Table S2). The scRNA-seq data from each study processed using R package Seurat. For cell-quality filtering, cells with < 200 expressed genes were excluded. The count values were log-normalized with a scale factor of 10,000. The top 2,000 variably expressed genes were identified using the FindVariableFeatures function. The ScaleData function were used to regress out unwanted sources of variation. Dimensionality was reduced via principal component analysis (PCA) incorporating highly variable features. To remove batch effects while preserving biological variation, R package harmony was used to integrate cells across individuals. Cell clusters were identified using the first 30 PCs with a clustering resolution of 0.8 via the FindClusters function. Cluster-specific genes were identified using the FindAllMarkers function, and the clusters were annotated by canonical cell markers.

Lodi *et al.* presented a single-cell atlas by aggregated 230 treatment-naive samples across 9 cancer types (Pan-cancer, 2025Lodi).^[1]^ We obtained the data from <https://lambrechtslab.sites.vib.be/en/dataaccess,> and reserved seven cancer type (NSCLC, BC, CRC, GBM, HCC, HNSC, and MEL). We identified clusters for 100,076 cancer/epithelial cells by Seurat, and grouped which into high and low subpopulations according to median value.

Salcher *et al.* aggregated 29 publicly available datasets to construct a NSCLC single-cell atlas (NSCLC, CoreAtlas).^[2]^ We downloaded the scRNA-seq data with cell type annotation information from Zenodo (https://doi.org/10.5281/zenodo.6411867). Neutrophils were extracted and annotated subpopulations according to the canonical markers.^[3]^

Additionally, we collected 15 scRNA-seq data of ICI immunotherapy pre-treatment for cancer samples (Table S3). RScore were computed for cells that were obtained from samples prior to immunotherapy. Cells derived from CR or PR patients were considered as response group, which derived from SD or PD patients were considered as non-response group.

**1.2 Spatial transcriptome RNA sequencing data processing**

Three publicly available spatial transcriptome RNA sequencing (stRNA-seq) datasets were obtained (Table S4). [Jenkins](https://pubmed.ncbi.nlm.nih.gov/?size=200&term=Jenkins+BH&cauthor_id=39757146) *et al*. employed 10x Genomics Visium workflow for immune-hot and immune-cold HNSC samples.^[4]^ Zhang *et al.* generated stRNA-seq data for seven HCC patients after anti-PD-1 therapy using 10x Genomics Visium platform.^[5]^ We processed the 10x Visium data using the Seurat package. Counts were first aligned to the tissue image to distinguish technical noise from true histological signals. Necrotic areas and tissue folding spots were then excluded, followed by normalization with the SCTransform function. RScore values were then calculated for each spot. Feng *et al.* performed spatial enhanced resolution omics-sequencing (Stereo-seq) on surgical CRC tissues from neoadjuvant PD-1 antibody treatment.^[6]^ The GEM files were processed using Stereopy, converted to H5ad format, and subsequently transformed into RDS objects for RScore calculation. Spot annotations were also obtained from Feng *et al.*

**1.3 Ro/e analysis**

Cells in scRNA-seq datasets were grouped into high RScore and low RScore groups by the median. We calculated the ratio of observed to expected (Ro/e) cell numbers for each annotated cell type in high RScore group among different samples by R package Startrac.^[7]^ An Ro/e value greater than one indicates the enrichment of a cell type in high RScore group.

**1.4 Cell-cell communication analysis**

Among pan-cancer scRNA-seq datasets, epithelial cells were grouped into high and low subpopulations according to the median of RScore. Cell-cell communications were inferred using R package CellChat.^[8]^ We focused on the human database in CellChat, identified over-expressed ligands or receptors by identifyOverExpressedGenes and identifyOverExpressedInteractions functions. Communication probabilities and cellular communication networks were computed and inferred using the computeCommunProb and filterCommunication functions (min.cells = 10). The computeCommunProbPathway and aggregateNet functions were used to infer the cell-cell communication at the signaling pathway level between each cell type. CellChat infers significant cell-cell communication by assigning probability values to each interaction and performing permutation tests. Due to huge number of cells, we randomly sample 2,000 epi-high and epi-low subpopulations respectively to infer cell-cell communications for 10 times in 2025Lodi dataset, the final communications were average.

**1.5 Functional analysis**

DEGs between high RScore and low RScore cells were identified using FindMarkers function in Seurat. R package clusterProfiler was used to KEGG function enrichment for top 100 upregulated DEGs.^[9]^ Pathways with *P* value < 0.05 were considered statistically significant.

The functional gene sets about CD8^+^ T, CD4^+^ T and B cells were acquired from Lodi *et al.* The genes for ISGscore nanostring obtained from Pelka *et al.* Signature of pre germinal center B and germinal center B are from Yang *et al.* and Ma *et al*. We evaluated the functional scores for cells using R package AUCell.^[10]^ Tian *et al.* defined (∑GPAGs-∑PPAGs) as a vessel normalization indicator. we evaluated vessel normalization level for endothelial cells. All the above gene sets are listed in Table S4.

The inferred CD8^+^ T cells infiltration values for TCGA samples by CIBERSORT, EPIC, and TIMER methods were downloaded from the TIMER2.0 (http://timer.comp-genomics.org/timer/) website.

**1.6 Receiver operating characteristic curve analysis**

To evaluate the predictive performance of the RScore signature, receiver operating characteristic (ROC) curve analysis was performed using R package pROC. The area under the ROC curve (AUC) was calculated to quantify the discriminative ability of RScore in distinguishing responders from non-responders. An AUC value closer to 1 indicates higher predictive accuracy.

**1.7 Survival analysis**

Samples were stratified based on the median of scores, and differences in survival outcomes were assessed using the log-rank test. Kaplan-Meier plots utilized to visualize the results. Survival analysis was conducted using R packages survival and survminer.

**1.8 Immunofluorescence staining**

Harvested mouse tumor tissues were fixed in 4% paraformaldehyde for 24 h, embedded in paraffin, and sectioned. Slides were deparaffinized, rehydrated, and subjected to high-temperature antigen retrieval and blocking. Sections were incubated overnight at 4°C with primary antibodies: anti-cleaved caspase-3 (Asp175) (1:500; Proteintech, Cat# 82707-13-RR) or anti-Granzyme B (1:1000; Selleck, Cat# A5122). After washing with PBS, sections were incubated with Fluorescein (FITC)–conjugated Goat Anti-Rabbit IgG(H+L) (1:200; Proteintech, Cat# SA00003-2) for 1 h at room temperature in the dark. Nuclei were counterstained with DAPI. Images were acquired using a Nikon Ts2 fluorescence microscope.

**1.9 Flow cytometry analysis of tumor-infiltrating lymphocytes**

Excised subcutaneous tumors were minced and digested in a solution containing 1 mg/mL Collagenase Type IV (Gibco, Cat# 17104019), 0.1 mg/mL Hyaluronidase (Thermo Scientific Chemicals, Cat# J62406), and 20 µg/mL DNase I (Gibco, Cat# 18047019) at 37°C to obtain single-cell suspensions. Following filtration through a cell strainer, cells were first stained with Fixable Viability Dye eFluor 506 (Thermo Fisher Scientific, Cat# 65-0866-14) for 15 min at 4°C in the dark to exclude dead cells. Subsequently, surface staining was performed by incubating cells with an antibody cocktail containing PerCP anti-mouse CD45 (Clone 30-F11; BioLegend, Cat# 103131), Pacific Blue anti-mouse CD3 (Clone 17A2; BioLegend, Cat# 100214), and PE anti-mouse CD8 (Clone 53-6.7; BioLegend, Cat# 100707) for 30 min at 4°C. After surface labeling, cells were fixed with 4% paraformaldehyde for 15 min at 4°C and permeabilized using Permeabilization Buffer (Proteintech, Cat# PK10002). Finally, intracellular staining was conducted by incubating cells with FITC anti-human/mouse Granzyme B (Clone GB11; BioLegend, Cat# 515403) for 30 min at 4°C in the dark.

**1.10 Flow cytometry of cocultured T cells**

T cells harvested from the co-culture system were first incubated with Zombie Red Fixable Viability Kit (BioLegend, Cat# 423109) for 15 min at 4°C to assess viability. This was followed by surface staining with PerCP anti-Human CD8 (Clone SK1; BioLegend, Cat# 344713) for 30 min at 4°C. After fixation and permeabilization as described above, intracellular Granzyme B was detected by incubating cells with FITC anti-human/mouse Granzyme B (Clone GB11; BioLegend, Cat# 515403) for 30 min at 4°C in the dark. All flow cytometry data were acquired using a Beckman CytoFLEX flow cytometer (Beckman Coulter) and analyzed using FlowJo software (v 10.8.1).

2. Supplemental Figures

## Figure S1. The ranked gene list for pan-cancer*.*

**(A)** Genes detected in at least five cancer types were ranked by their mean weight coefficient across cancer types. The weight coefficient reflects the contribution of each gene to immunotherapy response (responder vs. non-responder). Genes previously reported as immunotherapy response signatures in other studies are highlighted in color.

## Figure S2. Association of the RScore with immunotherapy response and patient survival

**(A)** The area under curve (AUC) values of RScores for predicting immunotherapy response across 25 bulk RNA-seq datasets. **(B)** The -log(P) values from one-tailed Wilcoxon rank-sum tests comparing RScore between responder and non-responder groups across 25 bulk RNA-seq datasets. **(C)-(K)** Survival analysis for patients stratified by high and low RScores in immunotherapy datasets based on overall survival (OS) data. **(L)-(R)** Survival analysis for patients stratified by high and low RScores in immunotherapy datasets based on progression-free survival (PFS) data.

## Figure S3. Spatial distribution of RScore in stRNA-seq datasets

**(A)** Distribution of RScores across spatial spots in immune-hot and immune-cold head and neck squamous cell carcinoma (HNSC) samples. **(B)** Comparison of RScores between immune-hot and immune-cold spots across all HNSC samples. **(C)** Distribution of RScores across spatial spots in responder and non-responder [hepatocellular carcinoma](https://www.google.com/search?q=Hepatocellular+carcinoma&sca_esv=3c51e872d41103f9&sxsrf=ANbL-n5di8sT8_6bNpFqrGoo-2J2h_0sXA:1771841867609&ei=SymcaeHuJOakqtsP1pOXwAc&biw=2195&bih=1033&ved=2ahUKEwiIiamqs--SAxUOmSYFHSq-C-cQgK4QegYIAQgAEAM&uact=5&oq=hcc+cncer&gs_lp=Egxnd3Mtd2l6LXNlcnAiCWhjYyBjbmNlcjIKEAAYgAQYChjLATIHEAAYgAQYDTIGEAAYDRgeMgYQABgNGB4yBhAAGA0YHjIGEAAYDRgeMgYQABgNGB4yBhAAGA0YHjIGEAAYDRgeMggQABgKGA0YHkjMKVAAWJomcAB4AZABAJgBjgWgAcokqgEFNC00LjW4AQPIAQD4AQL4AQGYAgmgAp0lwgIIEAAYgAQYywHCAgQQIxgnwgIKECMYgAQYJxiKBcICCxAuGIAEGNEDGMcBwgIFEAAYgATCAgoQABiABBhDGIoFwgIQEC4YgAQY0QMYQxjHARiKBcICBRAuGIAEwgILEC4YgAQYxwEYrwHCAgcQABiABBgMwgIIEC4YgAQYywHCAg4QLhiABBjHARjLARivAcICBBAAGB7CAgUQABjvBZgDAJIHBTQtNy4yoAeyWLIHBTQtNy4yuAedJcIHBTItNS40yAdDgAgA&sclient=gws-wiz-serp) (HCC) samples. **(D)** Comparison of RScore values between responder and non-responder spots across all HCC samples. **(E)** Distribution of Cell types and RScores across spatial spots in responder and non-responder colorectal cancer (CRC) samples. **(F)** Comparison of RScore values between responder and non-responder spots across all CRC samples.

## Figure S4. Distribution of nFeature_RNA and variation of RScores in pan-cancer scRNA-seq datasets*.*

**(A)** Proportion of cell types in pan-cancer scRNA-seq datasets. **(B)** Correlation of RScores calculated at different topN thresholds across cells from pan-cancer scRNA-seq datasets. **(C)** Distribution of nFeature_RNA stratified by quartiles in pan-cancer scRNA-seq datasets.

## Figure S5. KEGG functional enrichment analysis*.*

(**A**) KEGG functional enrichment analysis of genes upregulated in high RScore cells compared to low RScore cells.

## Figure S6. Annotation of cell subtypes and bulk RNA-seq deconvolution

(**A**) Correlation between functional scores and RScores in CD4 T cells. **(B, C)** Identification of cell clusters by Seurat and annotation of B cell subtypes in GSE131907 dataset. **(D, E)** Identification of cell clusters by Seurat and annotation of macrophage subtypes in OMIX005710 dataset. **(F)** Differential distribution of RScore between M1 and M2 macrophages. **(G)-(J)** The expression pattern of inflammatory caner associated fibroblast (iCAF) markers (CXCL14, PLAT, BMP4, and CFD). **(K)** Differential distribution of responder (R) and non-responder (NR) samples between high fib-RScore and low fib-RScore groups in the IMvigor210 dataset. **(L)** Survival analysis for high and low fib-RScore patients. Fibroblast expression was inferred using CIBERSORTx, the RScore of fibroblasts (fib-RScore) were calculated via scResponse.

## Figure S7. Annotation of neutrophil subtypes

**(A, B)** Identification of cell clusters by Seurat and annotation of neutrophil subtypes in CoreAtlas dataset. **(C)-(F)** Correlation between RScores and interferon‑stimulated gene (ISG) scores in neutrophils.

## Figure S8. Correlation of expression of ECSCR and COL4A1 with RScore and immunotherapy response

(**A**) Correlation of RScores and the expression of ECSCR (a good-prognosis angiogenesis genes, GPAG) in several cell types in GSE164690 dataset. **(B)** Correlation of RScores and the expression of COL4A1 (a poor-prognosis angiogenesis genes, PPAG) in several cell types in GSE164690 dataset. (C)-(D) Distribution of ECSCR^+^COL4A1^-^ and ECSCR^-^COL4A1^+^ cells between pathological complete response (pCR) and non-pCR patients in immunotherapy datasets (2023Li and 2024Ji). and (E) Distribution of ECSCR^+^COL4A1^-^ and ECSCR^-^COL4A1^+^ cells between responder (R) and non-responder (NR) in immunotherapy dataset 2024Shiao.

## Figure S9. Fibroblasts and monocytes/macrophages derived from responders exhibit high RScores*.*

**(A)-(C)** Fibroblasts in responsder (R) samples showed significantly higher RScores than those in non-responder (NR) samples across non-small cell lung cancer (NSCLC) and colorectal cancer (CRC) immunotherapy datasets. **(D)-(I)** Monocytes/Macrophages in R samples showed significantly higher RScores than those in NR samples across immunotherapy NSCLC, [hepatocellular carcinoma](https://www.google.com/search?q=Hepatocellular+carcinoma&sca_esv=3c51e872d41103f9&sxsrf=ANbL-n5di8sT8_6bNpFqrGoo-2J2h_0sXA:1771841867609&ei=SymcaeHuJOakqtsP1pOXwAc&biw=2195&bih=1033&ved=2ahUKEwiIiamqs--SAxUOmSYFHSq-C-cQgK4QegYIAQgAEAM&uact=5&oq=hcc+cncer&gs_lp=Egxnd3Mtd2l6LXNlcnAiCWhjYyBjbmNlcjIKEAAYgAQYChjLATIHEAAYgAQYDTIGEAAYDRgeMgYQABgNGB4yBhAAGA0YHjIGEAAYDRgeMgYQABgNGB4yBhAAGA0YHjIGEAAYDRgeMggQABgKGA0YHkjMKVAAWJomcAB4AZABAJgBjgWgAcokqgEFNC00LjW4AQPIAQD4AQL4AQGYAgmgAp0lwgIIEAAYgAQYywHCAgQQIxgnwgIKECMYgAQYJxiKBcICCxAuGIAEGNEDGMcBwgIFEAAYgATCAgoQABiABBhDGIoFwgIQEC4YgAQY0QMYQxjHARiKBcICBRAuGIAEwgILEC4YgAQYxwEYrwHCAgcQABiABBgMwgIIEC4YgAQYywHCAg4QLhiABBjHARjLARivAcICBBAAGB7CAgUQABjvBZgDAJIHBTQtNy4yoAeyWLIHBTQtNy4yuAedJcIHBTItNS40yAdDgAgA&sclient=gws-wiz-serp) (HCC), bladder cancer (BLCA), breast cancer (BC), and CRC immunotherapy datasets.

## Figure S10. Differential cell-cell communications of high and low RScore epithelial cells with other cell types.

(**A**) Identification of epithelial cell clusters by Seurat and grouped them into high RScore and low RScore subpopulations. **(B)** KEGG functional enrichment analysis of the top 100 differentially expressed genes in epithelial cells with high RScore versus low RScore. **(C, D)** Cell-cell communications among high and low RScore epithelial cells, and other cell subtypes, depicted by circle plot edge widths reflecting communication count and strength. **(E, F)** Pathway signals that high and low RScore epithelial cells received or sent. **(G)** Significant pathway signal high or low RScore epithelial cells to CD8^+^ T subtypes. **(H)** Number of sell-cell communications between high or low RScore epithelial cells with other cell types in pan-cancer scRNA-seq datasets.

## Figure S11. Correlation of metabolic pathway and immunotherapy outcome and immunotherapy infiltration

**(A, B)** Upregulation of glyoxylate and dicarboxylate metabolism scores in responder in gastric cancer (GC) and melanoma (MEL) immunotherapy datasets. **(C)-(F)** Downregulation of retinol metabolism scores in responder in non-small cell lung cancer (NSCLC), [hepatocellular carcinoma](https://www.google.com/search?q=Hepatocellular+carcinoma&sca_esv=3c51e872d41103f9&sxsrf=ANbL-n5di8sT8_6bNpFqrGoo-2J2h_0sXA:1771841867609&ei=SymcaeHuJOakqtsP1pOXwAc&biw=2195&bih=1033&ved=2ahUKEwiIiamqs--SAxUOmSYFHSq-C-cQgK4QegYIAQgAEAM&uact=5&oq=hcc+cncer&gs_lp=Egxnd3Mtd2l6LXNlcnAiCWhjYyBjbmNlcjIKEAAYgAQYChjLATIHEAAYgAQYDTIGEAAYDRgeMgYQABgNGB4yBhAAGA0YHjIGEAAYDRgeMgYQABgNGB4yBhAAGA0YHjIGEAAYDRgeMggQABgKGA0YHkjMKVAAWJomcAB4AZABAJgBjgWgAcokqgEFNC00LjW4AQPIAQD4AQL4AQGYAgmgAp0lwgIIEAAYgAQYywHCAgQQIxgnwgIKECMYgAQYJxiKBcICCxAuGIAEGNEDGMcBwgIFEAAYgATCAgoQABiABBhDGIoFwgIQEC4YgAQY0QMYQxjHARiKBcICBRAuGIAEwgILEC4YgAQYxwEYrwHCAgcQABiABBgMwgIIEC4YgAQYywHCAg4QLhiABBjHARjLARivAcICBBAAGB7CAgUQABjvBZgDAJIHBTQtNy4yoAeyWLIHBTQtNy4yuAedJcIHBTItNS40yAdDgAgA&sclient=gws-wiz-serp) (HCC), and breast cancer (BC) immunotherapy datasets. **(G, H)** Positive correlation of glyoxylate and dicarboxylate metabolism scores and CD8^+^ T infiltration evaluated by EPIC and CIBERSORT in TCGA. (I)-(K) Negative correlation of retinol metabolism scores and CD8^+^ T infiltration evaluated by EPIC, CIBERSORT, and TIMER in TCGA.

## Figure S12. Glyoxylate combined with anti-PD-1 therapy significantly suppresses B16-F10 melanoma growth.

**(A)** Representative gating strategy for GZMB^+^ cells in the in vitro co-culture system. **(B)** Representative gross images of excised tumors from B16-F10 tumor-bearing mice (n = 5). **(C)** Tumor volume growth curves (n = 5). **(D)** Tumor weights at the endpoint (n = 5). **(E)** Representative gating strategy for GzmB^+^ cells in murine subcutaneous tumor samples. **(F, G)** Representative flow cytometry graphs and quantification of the percentage of GzmB^+^ cells among CD8^+^ T cells within tumor-infiltrating lymphocytes (n = 5). **(H)** Representative immunofluorescence images (scale bar, 50 µm) of GzmB and cleaved caspase-3 staining in tumor sections (n = 5). **(I, J)** Quantification of the percentages of GzmB^+^ and cleaved caspase-3^+^ cells (n = 5). Data are presented as Mean ± SD. Each dot in the bar graphs represents an individual subject. Statistical significance was determined by two-tailed Student's t-test. **P* < 0.05; ***P* < 0.01; ****P* < 0.001.

3. Supplemental Tables

## Table S1. Bulk transcriptomics data of cancer samples that prior to immunotherapy.

See Supplemental Tables file.

## Table S2. Pan-cancer scRNA-seq datasets.

See Supplemental Tables file.

## Table S3. ScRNA-seq data of cancer samples that prior to immunotherapy.

See Supplemental Tables file.

## Table S4. Single-cell spatial transcriptomic datasets.

See Supplemental Tables file.

## Table S5. Functional gene sets and cell markers.

See Supplemental Tables file.

4. Abbreviations

| **Abbreviations** | **Definitions** |
| --- | --- |
| apCAF | antigen-presenting CAF |
| AUC | area under curve |
| BC | breast cancer |
| BD | Bhattacharyya distance |
| BLCA | bladder cancer |
| CAF | caner associated fibroblast |
| CIBERSORT | cell-type identification by estimating relative subsets of RNA transcripts |
| CR | complete response |
| CRC | colorectal cancer |
| DC | dendritic cell |
| DEGs | differentially expressed gene |
| Ds | direction strength |
| EPIC | estimating the proportions of immune and cancer cells |
| ESCA | esophageal carcinoma |
| GBM | glioblastoma |
| GC | gastric cancer |
| GDMT | glyoxylate and dicarboxylate metabolism |
| GPAG | good-prognosis angiogenesis gene |
| GSVA | gene set variation analysis |
| HCC | [hepatocellular carcinoma](https://www.google.com/search?q=Hepatocellular+carcinoma&sca_esv=3c51e872d41103f9&sxsrf=ANbL-n5di8sT8_6bNpFqrGoo-2J2h_0sXA:1771841867609&ei=SymcaeHuJOakqtsP1pOXwAc&biw=2195&bih=1033&ved=2ahUKEwiIiamqs--SAxUOmSYFHSq-C-cQgK4QegYIAQgAEAM&uact=5&oq=hcc+cncer&gs_lp=Egxnd3Mtd2l6LXNlcnAiCWhjYyBjbmNlcjIKEAAYgAQYChjLATIHEAAYgAQYDTIGEAAYDRgeMgYQABgNGB4yBhAAGA0YHjIGEAAYDRgeMgYQABgNGB4yBhAAGA0YHjIGEAAYDRgeMggQABgKGA0YHkjMKVAAWJomcAB4AZABAJgBjgWgAcokqgEFNC00LjW4AQPIAQD4AQL4AQGYAgmgAp0lwgIIEAAYgAQYywHCAgQQIxgnwgIKECMYgAQYJxiKBcICCxAuGIAEGNEDGMcBwgIFEAAYgATCAgoQABiABBhDGIoFwgIQEC4YgAQY0QMYQxjHARiKBcICBRAuGIAEwgILEC4YgAQYxwEYrwHCAgcQABiABBgMwgIIEC4YgAQYywHCAg4QLhiABBjHARjLARivAcICBBAAGB7CAgUQABjvBZgDAJIHBTQtNy4yoAeyWLIHBTQtNy4yuAedJcIHBTItNS40yAdDgAgA&sclient=gws-wiz-serp) |
| HNSC | head and neck squamous cell carcinoma |
| iCAF | inflammatory CAF |
| ICB | immune checkpoint blockade |
| IFN | interferon |
| ISG | interferon‑stimulated gene |
| MAD | median absolute deviation |
| MEL | melanoma |
| MHC | major histocompatibility complex |
| NK | natural killer cell |
| NR | non‑responder |
| NSCLC | non-small cell lung cancer |
| OS | overall survival |
| PBMC | peripheral blood mononuclear cell |
| PCA | principal component analysis |
| pCR | pathological complete response |
| PD | progressive disease |
| PFS | progression-free survival |
| PPAG | poor-prognosis angiogenesis gene |
| PR | partial response |
| Pw | position weight |
| R | responder |
| RCC | renal cell carcinoma |
| RGL | ranked gene list |
| ROC | receiver operating characteristic |
| Ro/e | ratio of observed to expected |
| RScore | response score |
| RTM | retinol metabolism |
| scRNA-seq | single‑cell RNA sequencing |
| SD | standard deviation |
| stRNA-seq | spatial transcriptome RNA sequencing |
| Tcm | central memory T |
| Tem | effector memory T |
| Tex | exhausted T |
| Tex^prog^ | progenitor population |
| Tex^term^ | terminally exhausted |
| TIMER | tumor immune estimation resource |
| TME | tumor microenvironment |
| Tn | naïve T |

5. References

1.	F. Lodi, S. Vanmassenhove, D. Chen, B. Boeckx, L. Ferreira Maciel, F. Peeters, E. Donders, H. Song, L. Harbers, J. Pozniak, L. Loverix, Y. Zhou, M. Bila, A. Bassez, S. Cappuyns, T. Venken, S. Olbrecht, A. Franken, P. Van Mol, R. Schepers, T. van Brussel, G. Philips, H. Vos, M. Keupers, F. De Smet, S. Tejpar, O. Bechter, G. Bergers, A. Smeets, P. Clement, E. Wauters, J. Dekervel, T. Van Gorp, J. Qian, J. C. Marine, D. Lambrechts, "Decoding tumor heterogeneity: A spatially informed pan-cancer analysis of the tumor microenvironment" *Cell Rep Med* 2025, *6* (10), 102416, <https://doi.org/10.1016/j.xcrm.2025.102416>.

2.	S. Salcher, G. Sturm, L. Horvath, G. Untergasser, C. Kuempers, G. Fotakis, E. Panizzolo, A. Martowicz, M. Trebo, G. Pall, G. Gamerith, M. Sykora, F. Augustin, K. Schmitz, F. Finotello, D. Rieder, S. Perner, S. Sopper, D. Wolf, A. Pircher, Z. Trajanoski, "High-resolution single-cell atlas reveals diversity and plasticity of tissue-resident neutrophils in non-small cell lung cancer" *Cancer Cell* 2022, *40* (12), 1503, <https://doi.org/10.1016/j.ccell.2022.10.008>.

3.	L. Horvath, C. Puschmann, A. Scheiber, A. Martowicz, G. Sturm, Z. Trajanoski, D. Wolf, A. Pircher, S. Salcher, "Beyond binary: bridging neutrophil diversity to new therapeutic approaches in NSCLC" *Trends Cancer* 2024, *10* (5), 457, <https://doi.org/10.1016/j.trecan.2024.01.010>.

4.	B. H. Jenkins, I. Tracy, M. Rodrigues, M. J. L. Smith, B. R. Martinez, M. Edmond, S. Mahadevan, A. Rao, H. Zong, K. Liu, A. Aggarwal, L. Li, L. Diehl, E. V. King, J. G. Bates, C. J. Hanley, G. J. Thomas, "Single cell and spatial analysis of immune-hot and immune-cold tumours identifies fibroblast subtypes associated with distinct immunological niches and positive immunotherapy response" *Mol Cancer* 2025, *24* (1), 3, <https://doi.org/10.1186/s12943-024-02191-9>.

5.	S. Zhang, L. Yuan, L. Danilova, G. Mo, Q. Zhu, A. Deshpande, A. T. F. Bell, J. Elisseeff, A. S. Popel, R. A. Anders, E. M. Jaffee, M. Yarchoan, E. J. Fertig, L. T. Kagohara, "Spatial transcriptomics analysis of neoadjuvant cabozantinib and nivolumab in advanced hepatocellular carcinoma identifies independent mechanisms of resistance and recurrence" *Genome Med* 2023, *15* (1), 72, <https://doi.org/10.1186/s13073-023-01218-y>.

6.	Y. Feng, W. Ma, Y. Zang, Y. Guo, Y. Li, Y. Zhang, X. Dong, Y. Liu, X. Zhan, Z. Pan, M. Luo, M. Wu, A. Chen, D. Kang, G. Chen, L. Liu, J. Zhou, R. Zhang, "Spatially organized tumor-stroma boundary determines the efficacy of immunotherapy in colorectal cancer patients" *Nat Commun* 2024, *15* (1), 10259, <https://doi.org/10.1038/s41467-024-54710-3>.

7.	L. Zhang, X. Yu, L. Zheng, Y. Zhang, Y. Li, Q. Fang, R. Gao, B. Kang, Q. Zhang, J. Y. Huang, H. Konno, X. Guo, Y. Ye, S. Gao, S. Wang, X. Hu, X. Ren, Z. Shen, W. Ouyang, Z. Zhang, "Lineage tracking reveals dynamic relationships of T cells in colorectal cancer" *Nature* 2018, *564* (7735), 268, <https://doi.org/10.1038/s41586-018-0694-x>.

8.	S. Jin, C. F. Guerrero-Juarez, L. Zhang, I. Chang, R. Ramos, C. H. Kuan, P. Myung, M. V. Plikus, Q. Nie, "Inference and analysis of cell-cell communication using CellChat" *Nat Commun* 2021, *12* (1), 1088, <https://doi.org/10.1038/s41467-021-21246-9>.

9.	S. Xu, E. Hu, Y. Cai, Z. Xie, X. Luo, L. Zhan, W. Tang, Q. Wang, B. Liu, R. Wang, W. Xie, T. Wu, L. Xie, G. Yu, "Using clusterProfiler to characterize multiomics data" *Nat Protoc* 2024, *19* (11), 3292, <https://doi.org/10.1038/s41596-024-01020-z>.

10.	S. Aibar, C. B. Gonzalez-Blas, T. Moerman, V. A. Huynh-Thu, H. Imrichova, G. Hulselmans, F. Rambow, J. C. Marine, P. Geurts, J. Aerts, J. van den Oord, Z. K. Atak, J. Wouters, S. Aerts, "SCENIC: single-cell regulatory network inference and clustering" *Nat Methods* 2017, *14* (11), 1083, <https://doi.org/10.1038/nmeth.4463>.
